# Biallelic variants in *TRAPPC10* cause a microcephalic TRAPPopathy disorder in humans and mice

**DOI:** 10.1371/journal.pgen.1010114

**Published:** 2022-03-17

**Authors:** Lettie E. Rawlins, Hashem Almousa, Shazia Khan, Stephan C. Collins, Miroslav P. Milev, Joseph Leslie, Djenann Saint-Dic, Valeed Khan, Ana Maria Hincapie, Jacob O. Day, Lucy McGavin, Christine Rowley, Gaurav V. Harlalka, Valerie E. Vancollie, Wasim Ahmad, Christopher J. Lelliott, Asma Gul, Binnaz Yalcin, Andrew H. Crosby, Michael Sacher, Emma L. Baple

**Affiliations:** 1 RILD Wellcome Wolfson Medical Research Centre, RD&E (Wonford) NHS Foundation Trust, University of Exeter Medical School, Exeter, United Kingdom; 2 Peninsula Clinical Genetics Service, Royal Devon & Exeter Hospital (Heavitree), Exeter, United Kingdom; 3 Department of Biology, Concordia University, Montreal, Quebec, Canada; 4 Department of Biological Sciences, International Islamic University, Islamabad, Pakistan; 5 Institute of Genetics and Molecular and Cellular Biology, Inserm, Illkirch, France; 6 Inserm, University of Bourgogne Franche-Comté, Dijon, France; 7 Department of Molecular Diagnostics, Rehman Medical Institute, Peshawar, Pakistan; 8 Faculty of Health, University of Plymouth, Plymouth, United Kingdom; 9 University Hospitals Plymouth NHS Trust, Plymouth, United Kingdom; 10 Wellcome Sanger Institute, Hinxton, Cambridge, United Kingdom; 11 Department of Pharmacology, Rajarshi Shahu College of Pharmacy, Malvihir, Buldana, India; 12 Department of Biochemistry, Faculty of Biological Sciences, Quaid-i-Azam University, Islamabad, Pakistan; 13 Department of Anatomy and Cell Biology, McGill University, Montreal, Quebec, Canada; HudsonAlpha Institute for Biotechnology, UNITED STATES

## Abstract

The highly evolutionarily conserved transport protein particle (TRAPP) complexes (TRAPP II and III) perform fundamental roles in subcellular trafficking pathways. Here we identified biallelic variants in *TRAPPC10*, a component of the TRAPP II complex, in individuals with a severe microcephalic neurodevelopmental disorder. Molecular studies revealed a weakened interaction between mutant TRAPPC10 and its putative adaptor protein TRAPPC2L. Studies of patient lymphoblastoid cells revealed an absence of TRAPPC10 alongside a concomitant absence of TRAPPC9, another key TRAPP II complex component associated with a clinically overlapping neurodevelopmental disorder. The TRAPPC9/10 reduction phenotype was recapitulated in *TRAPPC10*^*-/-*^ knockout cells, which also displayed a membrane trafficking defect. Notably, both the reduction in TRAPPC9 levels and the trafficking defect in these cells could be rescued by wild type but not mutant TRAPPC10 gene constructs. Moreover, studies of *Trappc10*^*-/-*^ knockout mice revealed neuroanatomical brain defects and microcephaly, paralleling findings seen in the human condition as well as in a *Trappc9*^*-/-*^ mouse model. Together these studies confirm autosomal recessive *TRAPPC10* variants as a cause of human disease and define TRAPP-mediated pathomolecular outcomes of importance to TRAPPC9 and TRAPPC10 mediated neurodevelopmental disorders in humans and mice.

## Author summary

Microcephalic neurodevelopmental disorders are a group of conditions that are often inherited in families, involving small head size and abnormal brain development and function. This often results in delayed development of an affected child, affecting their movement, language and/or non-verbal communication and learning, as well as seizures and neuropsychiatric problems. A group of proteins called the transport protein particles (TRAPPs) are important for the transport of cargos inside cells. Alterations within a number of the TRAPP proteins have previously been associated with human inherited diseases called the ‘TRAPPopathies’, which involve neurodevelopmental and skeletal abnormalities. Here we show that *TRAPPC10* gene alterations cause a new TRAPPopathy microcephalic neurodevelopmental disorder, and we provide a detailed clinical description of the condition termed ‘*TRAPPC10*-related disorder’. Our studies in mice lacking the *TRAPPC10* gene identified similar features to those of affected humans, including small brain size and skeletal abnormalities. Our molecular studies showed that an affected individual with an alteration in the *TRAPPC10* gene has no functional TRAPPC10 protein in their cells, which in turn causes a reduction in levels of another important TRAPP molecule, TRAPPC9. Cells lacking TRAPPC10 also display abnormalities in cellular transport processes. Together our data confirm alterations in *TRAPPC10* as a cause of a microcephalic neurodevelopmental disorder in both humans and mice.

## Introduction

The transport protein particles (TRAPPs) comprise highly evolutionarily conserved multiprotein complexes, originally identified in yeast. Three yeast TRAPP complexes have been described (TRAPP I, II and III) which play important roles in secretory and endocytic subcellular trafficking pathways [1,2]. Humans possess only two complexes (TRAPP II and III), both sharing the same core proteins alongside additional complex-specific components, some not identified in yeast. Previous studies have identified diverse cellular roles for the TRAPP II complex including Golgi, COP (coat protein)-I vesicular, lipid droplet, centrosomal and ciliary functions [3–5]. The TRAPP II complex contains two complex-specific subunits; TRAPPC9 and TRAPPC10 [6]. An additional TRAPP II complex-associated protein has recently been described (TRAPPC14/C7orf43) [7]. However, TRAPPC14 does not appear to be required for TRAPP II complex-associated guanine nucleotide exchange factor activity [8,9] and lack of the molecule does not affect TRAPP II complex assembly or stability [7]. Thus, further studies are required to clarify the role of TRAPPC14 in TRAPP II. TRAPPC9 has been implicated in COP II vesicle trafficking [10] and NF-κB signalling pathway activation [11], with TRAPPC9 depletion associated with increased lipid droplet formation [4]. TRAPPC14 plays a role in Rabin 8 binding to the TRAPP II complex and its tethering of preciliary vesicles to the mother centriole during ciliogenesis [7]. No detailed studies of TRAPPC10 function have been conducted, although it has been shown to interact with TRAPPC2L and may have roles in guanine nucleotide exchange factor (GEF) activity regulation [12].

Ten of the 15 TRAPP complex proteins have been associated with human inherited diseases, collectively termed “TRAPPopathies” [6]. This includes TRAPPC2, the only subunit associated with skeletal dysplasia (spondyloepiphyseal dysplasia tarda (SEDT); OMIM #313400) [13], and nine other TRAPP subunits (TRAPPC2L, TRAPPC4, TRAPPC6A, TRAPPC6B, TRAPPC9, TRAPPC10, TRAPPC11, TRAPPC12 and TRAPPC14) associated with neurodevelopmental disorders [12,14–24]. While diverse, the clinical features of these disorders are overlapping and include neurodevelopmental delay, intellectual disability, developmental regression, primary and postnatal-onset microcephaly, epilepsy, hypotonia, craniofacial dysmorphism and structural brain abnormalities including thinning of the corpus callosum and reduced white matter volume (S1 Table).

Of the TRAPP II complex specific subunits, only TRAPPC9 has been robustly associated with human disease, an autosomal recessive neurodevelopmental disorder (OMIM #613192) characterized by postnatal-onset microcephaly with reduced white matter volume and corpus callosum thinning, intellectual disability, dysmorphic features, hypotonia, epilepsy, and raised body mass index [18–20]. TRAPP II plays important roles in Golgi membrane trafficking. “Golgipathy” neurodevelopmental disorders including the TRAPPopathies comprise an expansive group of conditions associated with variants in Rab GTPases, conserved oligomeric Golgi (COG) complex, and coat protein (COP) complex proteins among others [25,26] and are characterized by primary or postnatal-onset microcephaly, intellectual disability, seizures and white matter brain abnormalities.

Here, we present comprehensive genetic, clinical, functional and mouse data to define biallelic *TRAPPC10* gene variants as a cause of a microcephalic neurodevelopmental disorder, providing important insight into the TRAPPopathy family of disorders.

## Results

### Biallelic *TRAPPC10* variants are associated with a microcephalic neurodevelopmental disorder

**Fig 1:**
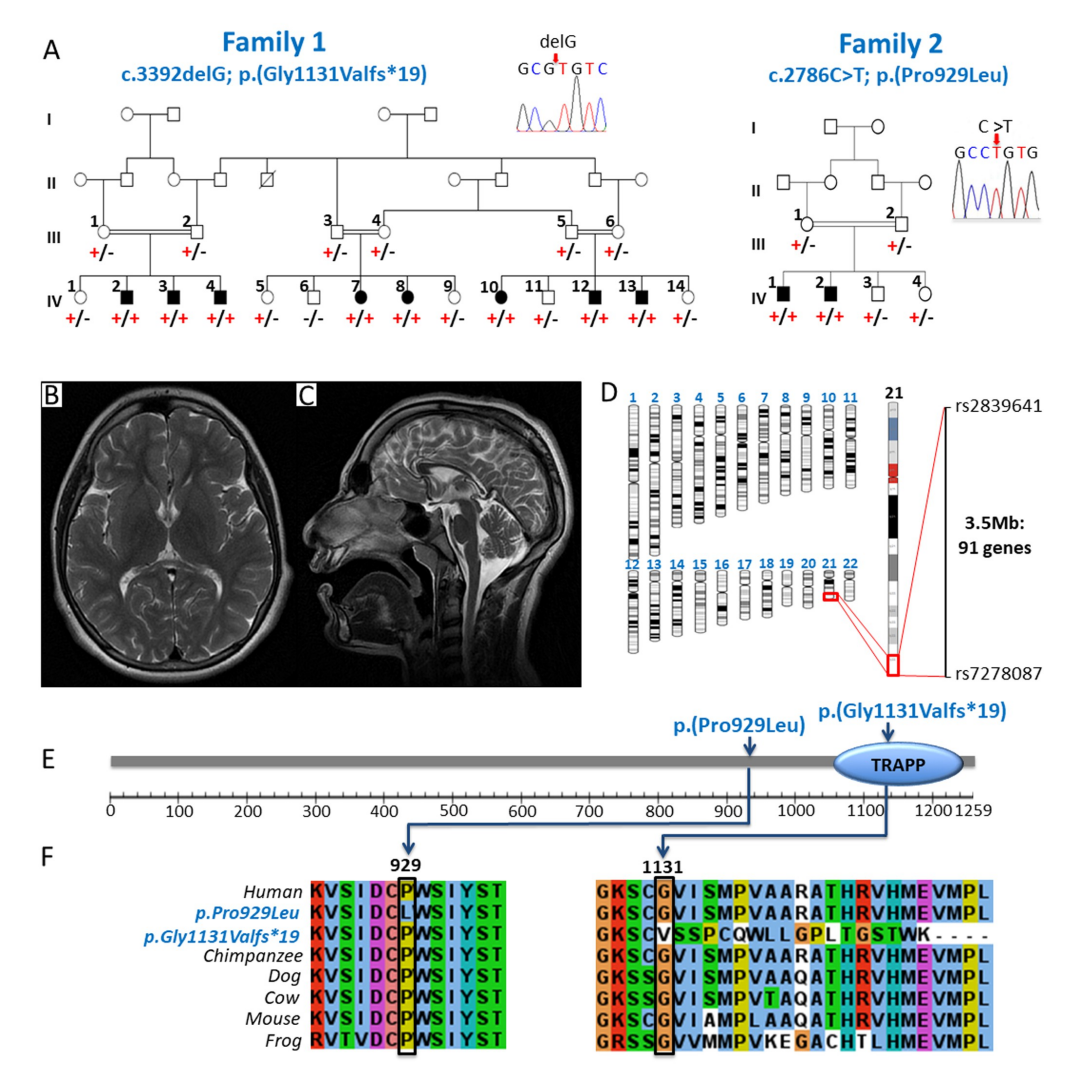
Biallelic *TRAPPC10* variants identified in two families with individuals affected by a microcephalic neurodevelopmental disorder. (**A**) **Family 1** Extended pedigree comprising three interlinking families and a total of eight affected individuals all of whom are homozygous for the *TRAPPC10* c.3392delG; p.(Gly1131Valfs*19) variant (‘+’), with co-segregation confirmed in other family members (‘-’ indicates the wild type allele). **Inset:** Dideoxy sequence chromatogram of an affected individual homozygous for the *TRAPPC10* c.3392delG; p.(Gly1131Valfs*19) variant. **Family 2** Previously reported family (MR107) [27] showing co-segregation of the *TRAPPC10* c.2786C>T; p.(Pro929Leu) variant (‘+’) in a four generation pedigree (‘-’ indicates the wild type allele). **Inset:** Dideoxy sequence chromatogram of an affected individual homozygous for the *TRAPPC10* c.2786C>T; p.(Pro929Leu) variant. (**B, C**) MRI neuroimaging of individual IV:2 from family 1 demonstrates microcephaly and profound thinning of the corpus callosum with no other abnormalities. (**D**) Genome-wide SNP mapping in seven affected individuals (1-IV:2–4, 1-IV:7–8 and 1-IV:12–13) identified a single (3.5Mb) region of shared homozygosity, containing 91 genes including *TRAPPC10*. (**E**) Schematic diagram of the TRAPPC10 protein identifying *TRAPPC10* sequence variants and TRAPP domain. (**F**) Multi-species alignment showing conservation of the molecular region encompassing the p.Gly1131Valfs*19 and p.Pro929Leu variants.

Table 1 summarizes the clinical features of affected individuals in this study. Family 1 comprises a large interlinking multi-nuclear Pakistani family, with eight individuals (4.1–18.1 years) affected by a severe microcephalic neurodevelopmental disorder (Fig 1A, Family 1). All but one individual presented with microcephaly (>3 standard deviations (SDS) below mean), and all displayed short stature (-1.78 to -5.09 SDS). All eight were born at full term, prenatal histories were unremarkable, although antenatal ultrasound scans were not performed. The parents did not report that any affected infant’s head was noticeably small (no weight or occipitofrontal circumference (OFC) measurements were recorded). The earliest OFC measurement documented was at 2.5 years (1-IV:4), and at age 4.1 years his microcephaly had not worsened. Affected individuals displayed mild craniofacial dysmorphism (S1A–S1C Fig). Although standardized intelligence quotient (IQ) testing was not possible due to cultural and language barriers, all affected individuals were assessed as severely intellectually impaired (DSM-5 criteria). Developmental trajectories were similar in all, as was the degree of impairment across all developmental domains. Hypotonia in infancy was universal and walking was delayed to between 2.5–4 years. All affected individuals have speech impairment, five have only a few words and three are non-verbal. Behavioural abnormalities including autistic features and aggressive episodes were a consistent feature. A history of seizures from early infancy was reported in four individuals, one has ongoing generalised tonic-clonic seizures (1-IV:2) and the remaining three (1-IV:3, 1-IV:7, 1-IV:13) are seizure free on medication. Neuroimaging was only performed for one case (1-IV:2) due to the remote family location and as sedation/general anaesthesia was deemed necessary. This revealed microcephaly with corpus callosum thinning and no other abnormalities (Fig 1B and 1C). All had normal systemic examination findings and no evidence of gross skeletal disproportion (S1A Fig). It was only possible to obtain skeletal radiographs for a single individual (1-IV:12) aged 9 years, these were unremarkable but unfortunately spinal imaging was incomplete.

**Table 1:** A comparison of clinical features of affected individuals with biallelic variants in *TRAPPC10*.

FAMILY	FAMILY 1								FAMILY 2	
Pedigree reference	IV:2	IV:3	IV:4	IV:7	IV:8	IV:10	IV:12	IV:13	IV:1	IV:2
Genotype	p.(G1131Vfs*19)/ p.(G1131Vfs*19)	p.(G1131Vfs*19)/ p.(G1131Vfs*19)	p.(G1131Vfs*19)/ p.(G1131Vfs*19)	p.(G1131Vfs*19)/ p.(G1131Vfs*19)	p.(G1131Vfs*19)/ p.(G1131Vfs*19)	p.(G1131Vfs*19)/ p.(G1131Vfs*19)	p.(G1131Vfs*19)/ p.(G1131Vfs*19)	p.(G1131Vfs*19)/ p.(G1131Vfs*19)	p.(P929L)/ p.(P929L)	p.(P929L)/ p.(P929L)
Gender	M	M	M	F	F	F	M	M	M	M
Age at evaluation (years)	18.1	13.8	4.1	14.1	15.6	5.5	9.0	13.7	25	22
GROWTH										
Birth weight kg (SDS)	NK	NK	2.5 (-2.25)	NK	NK	NK	NK	NK	NK	NK
OFC cm (SDS)	55 (-1.32)	50 (-3.49)	47 (-3.59)	46 (-6.64)	49 (-4.56)	43 (-7.46)	43 (-7.05)	47 (-5.28)	50 (-4.24)	53 (-2.49)
Height cm (SDS)	164 (-1.87)	146 (-1.78)	94 (-2.23)	127 (-5.09)	142 (-3.41)	99 (-2.78)	119 (-2.48)	125 (-4.26)	150 (-3.92)	140 (-5.29)
Weight kg (SDS)	52 (-1.96)	40 (-0.99)	18 (0.6)	25 (-4.9)	45 (-1.39)	16 (-1.44)	20 (-2.77)	28 (-3.16)	45 (-3.9)	38 (-4.87)
BMI	19.3	18.7	20.4	15.5	22.3	16.3	14.1	17.9	20	19
DEVELOPMENT										
Intellectual disability	Severe	Severe	Severe	Severe	Severe	Severe	Severe	Severe	Severe	Severe
Global developmental delay	✓	✓	✓	✓	✓	✓	✓	✓	✓	✓
Speech impairment	✓ <10 words	✓ Non-verbal	✓ <10 words	✓ Non-verbal	✓ <10 words	✓ Non-verbal	✓ <10 words	✓ <10 words	✓	✓
Walked (years)	4	4	Standing	3.5	2.5	4	4	3	NK	NK
Hearing	No concerns	Otitis media	No concerns	No concerns	No concerns	No concerns	No concerns	No concerns	No concerns	No concerns
Vision	No concerns	No concerns	No concerns	No concerns	No concerns	No concerns	Strabismus	No concerns	Strabismus	Strabismus
NEUROLOGY										
Childhood hypotonia	✓	✓	✓	✓	✓	✓	✓	✓	NK	NK
Seizures	✓	✓	-	✓	-	-	-	✓	-	-
Gait abnormalities	-	Waddling gait	-	-	-	-	Waddling gait	Waddling gait	NK	NK
Behavioural abnormalities	✓	✓	✓	✓	✓	✓	✓	✓	✓	✓

Abbreviations: M, male; F, female; NK, not known; SDS, standard deviation score; OFC, occipitofrontal circumference; G, glycine; V, valine; fs, frameshift; (✓), indicates presence of a feature in an affected individual; (-), indicates absence of a feature in an affected individual. Height, weight and OFC Z-scores were calculated using a Microsoft Excel add-in to access growth references based on the LMS method^a^ using UK 1990 reference population^b^

^a^ Pan H, Cole TJ. LMS growth, a Microsoft Excel add-in to access growth references based on the LMS method. Version 2.77, http://www.healthforallchildren.co.uk/;2012

^b^ Cole TJ, Freeman JV, Preece MA: British 1990 growth reference centiles for weight, height, body mass index and head circumference fitted by maximum penalized likelihood. Stat Med 1998, 17(4):407–4.

Assuming homozygosity for a founder variant was responsible, we undertook SNP genotyping (1-IV:2–4, 1-IV:7–8, 1-IV:12–13) in parallel with exome sequencing (1-IV:2) to identify the cause of disease. SNP mapping identified a single notable (>1Mb) homozygous region common to all affected individuals (3.5Mb, rs2839641-rs7278087; chr21:g.43,179,611–46,678,912 [hg38]) as the likely disease locus (Fig 1D). Concomitant exome sequencing data analysis to identify candidate homozygous and compound heterozygous rare (<0.01 frequency) variants predicted to have a functional impact, identified a single candidate variant within this region which could not be excluded. This frameshift variant (chr21:g.44102823del; NM_003274.4:c.3392del; p.(Gly1131Valfs*19)[hg38]; Fig 1A) in the penultimate (22/23) exon of *TRAPPC10* located in the chromosome 21q22.12 region, is present within the C-terminal TRAPP domain (Fig 1E) and is predicted to result in nonsense mediated mRNA decay of the mutant transcript (Fig 1F). The variant was confirmed in the lymphocyte RNA of an affected individual (S2 Fig), is not listed in publicly available genomic databases (including gnomAD v2.1.1/v3.1.1) and co-segregates appropriately (Fig 1A).

This finding enabled us to revisit the genetic data and more comprehensively phenotype two siblings in an unrelated Pakistani family (Fig 1A, Family 2) included in a larger study to identify novel candidate neurodevelopmental disease genes ([27] Family MR107). Both male siblings (2-IV:1, 22 years and 2-IV:2, 25 years), were described to have “severe intellectual disability, aggressive behaviour and poor speech”. A homozygous exon 18 *TRAPPC10* variant (chr21:g.44089849C>T; NM_003274.4:c.2786C>T; p.(Pro929Leu) [hg38]) (Fig 1A) was identified as a potential cause of disease [27]. This variant, which co-segregated appropriately, is absent from gnomAD v2.1.1/v3.1.1. Multiple species alignment confirms stringent conservation of Pro929 across vertebrates (Fig 1F). Additional clinical features noted and not previously reported, included microcephaly/borderline microcephaly (-2.49 SDS, -4.2 SDS), short stature (-3.86,– 5.29 SDS) and mild dysmorphism, all consistent with Family 1.

### TRAPPC10 variants exhibit reduced interaction with TRAPPC2L, destabilize the TRAPP II complex and result in an anterograde trafficking defect

**Fig 2:**
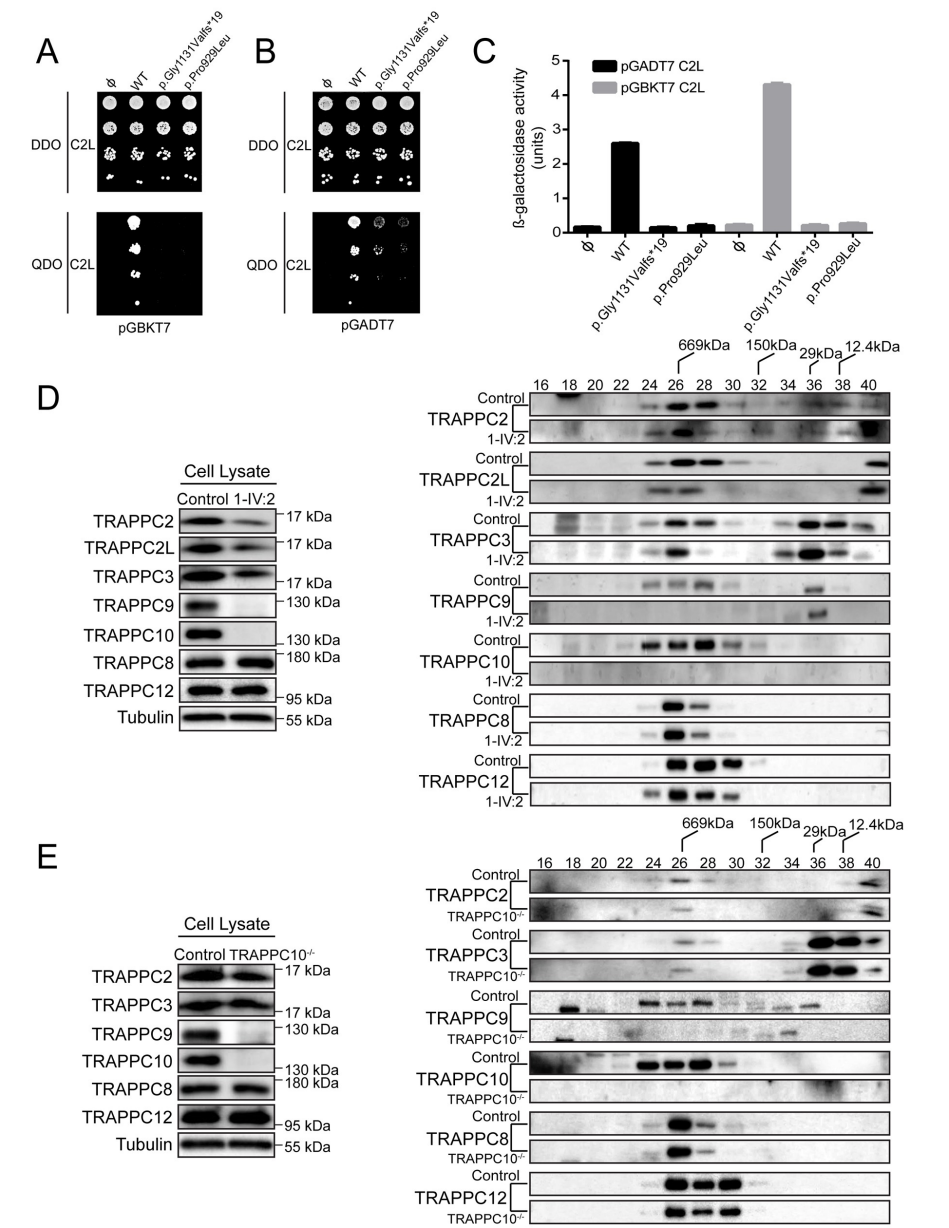
TRAPP II complex integrity is affected by TRAPPC10 variants and in TRAPPC10^-/-^ cells. Yeast cells transformed with pGBKT7 or pGBKT7 expressing either wild type TRAPPC10, the p.Gly1131Valfs*19 variant or the p.Pro929Leu variant (**A**), or pGADT7 or pGADT7 expressing either wild type TRAPPC10, the p.Gly1131Valfs*19 variant or the p.Pro929Leu variant (**B**) were mated with yeast expressing either pGBKT7-TRAPPC2L (**A**) or pGADT7-TRAPPC2L (**B**). Serial dilutions of the diploid cells were grown on SC medium lacking leucine and tryptophan (DDO) or SC medium lacking leucine, tryptophan, histidine and adenine (QDO). (**C**) Diploid cells from (**A**) and (**B**) were quantitatively tested for β-galactosidase activity. Units of activity were calculated according to the following formula: (OD_420_ x 1000)/(OD_600_ x time in hours). Note that western analysis revealed that all forms of TRAPPC10 and the two variants were expressed in the yeast cells. (**D**) Lymphoblastoid cells from control or an individual homozygous for the TRAPPC10 p.(Gly1131Valfs*19) variant were lysed and probed for the TRAPP proteins indicated and for tubulin as a representative loading control. The lysates were fractionated on a Superose 6 size exclusion column. Fractions of 0.5ml were collected. Neighbouring fractions were pooled and fractionated by SDS-PAGE and probed for the indicated TRAPP proteins. (**E**) Wild type HEK293 or TRAPPC10^-/-^ cells were lysed and probed for the TRAPP proteins indicated and for tubulin as a representative loading control. The lysates were fractionated on a Superose 6 size exclusion column. Fractions of 0.5ml were collected. Neighbouring fractions were pooled and fractionated by SDS-PAGE and probed for the indicated TRAPP proteins. The fractionation of molecular size standards are indicated above the top-most panel of the size exclusion portion of (**D**) and (**E**).

We next sought to determine the molecular consequences of each *TRAPPC10* genetic variant. As we previously determined that TRAPPC10 interacts with TRAPPC2L in the TRAPP II complex [12], we used yeast two-hybrid assays to investigate how TRAPPC10 alteration affects TRAPPC2L interactions. When TRAPPC10 was in the bait (pGBKT7) or prey (pGADT7) vector, TRAPPC2L interaction was observed (Fig 2A and 2B). However, both p.Gly1131Valfs*19 and p.Pro929Leu variants showed reduced TRAPPC2L interaction (Fig 2A and 2B), though the proteins were expressed in the yeast cells (not shown). This was confirmed quantitatively by measuring β-galactosidase activity (Fig 2C), one of the yeast two-hybrid system markers. Given this, we generated a patient lymphoblastoid cell line (LCL) (1-IV:2; *TRAPPC10* p.(Gly1131Valfs*19)) to investigate TRAPP complex outcomes. We examined core TRAPP protein (TRAPPC2, TRAPPC2L, TRAPPC3) levels, as well as TRAPP III-specific (TRAPPC8, TRAPPC12), and TRAPP II-specific (TRAPPC10, TRAPPC9) levels. TRAPPC2L, TRAPPC2 and TRAPPC3 levels were slightly reduced in the p.(Gly1131Valfs*19) lysate compared to control (Fig 2D). As expected we did not detect TRAPPC10 as the commercially available TRAPPC10 antibody is directed to the molecular region downstream (C-terminal) to the p.(Gly1131Valfs*19) alteration. Intriguingly we also noted an absence of full length TRAPPC9, although in contrast, TRAPP III-specific proteins levels were not affected. We then fractionated p.(Gly1131Valfs*19) and control LCL lysates on a size exclusion column. While TRAPPC10 and TRAPPC9 fractionated as expected in controls, they were absent from p.(Gly1131Valfs*19) lysate (Fig 2D) indicating an absence of TRAPP II (peak in fraction 28 of control cells). There was no change in core protein (TRAPPC2, TRAPPC2L, TRAPPC3) fractionation consistent with their presence in TRAPP III, although the portion that co-fractionates with TRAPP II was reduced. Similarly, no change in TRAPP III proteins (TRAPPC8, TRAPPC12) fractionation was seen, indicating that this complex was not affected in p.(Gly1131Valfs*19) cells.

**Fig 3:**
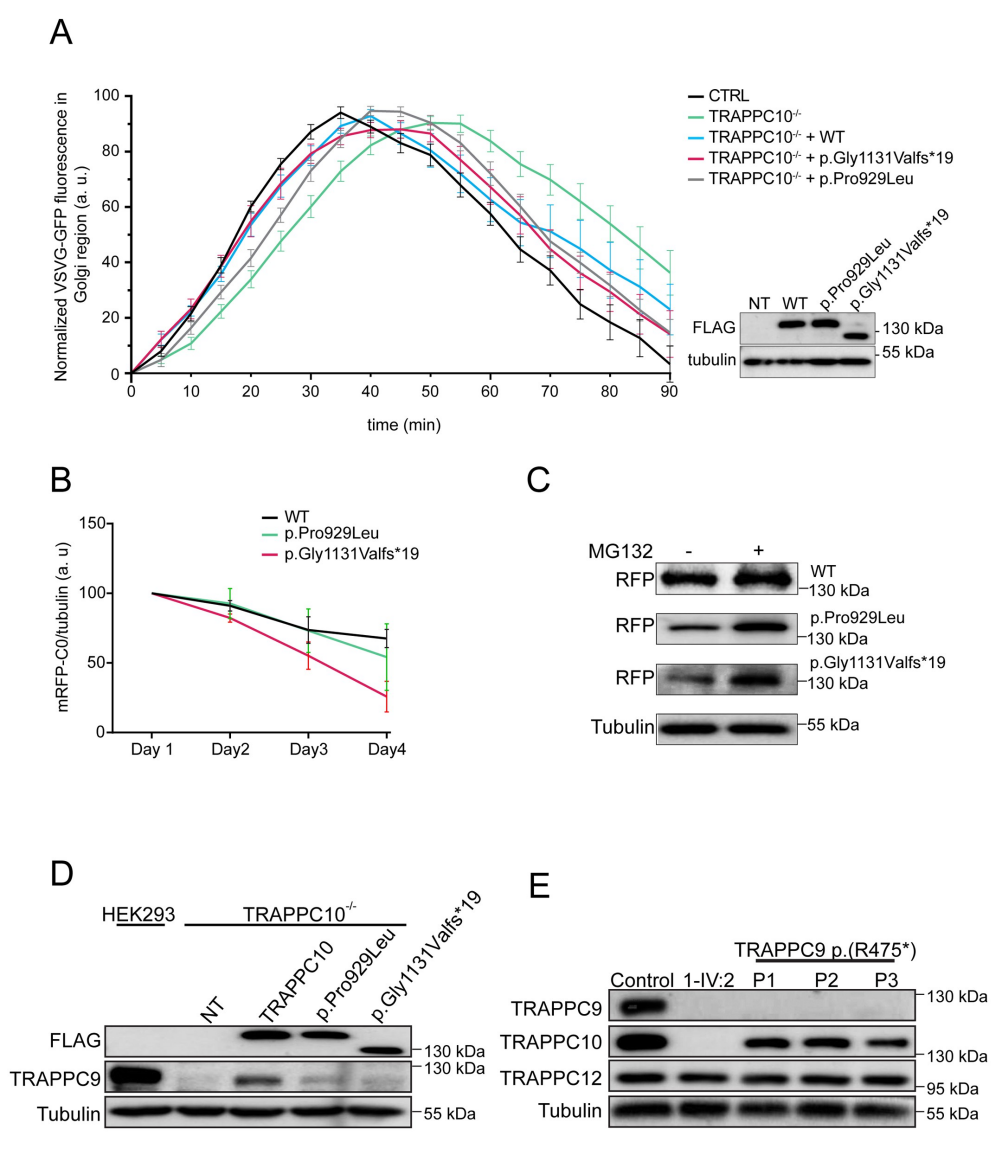
Cells devoid of TRAPPC10 display a membrane trafficking defect and lack detectable levels of TRAPPC9. (**A**) Wild type HEK293 cells or TRAPPC10^-/-^ cells either not transfected or transfected with FLAG-tagged wild type TRAPPC10 or one of the TRAPPC10 variants indicated were infected with VSVG-GFP ts045 4 hours after transfection. After an overnight incubation at 40°C, the cells were shifted to 32°C and imaged every minute. The fluorescence intensity in the Golgi was quantified and plotted versus time as described in the Materials and Methods section. The inset shows a western blot for the transfected proteins probed with anti-FLAG antibody. Representative images are shown in S7 Fig. (**B**) RFP-tagged versions of either wild type TRAPPC10, the p.Gly1131Valfs*19 variant or the p.Pro929Leu variant were transfected into HeLa cells and lysates prepared every day for 4 days. The lysates were fractionated by SDS-PAGE and probed with anti-mCherry antibody to reveal the RFP-tagged construct. Though the trends were consistent, statistical significance was seen only at day 4. The half-life for each overexpressed protein was found to be approximately 5.4 days, 4.4 days and 3.1 days for the wild type, p.Gly1131Valfs*19 and p.Pro929Leu variants, respectively. (**C**) RFP-tagged TRAPPC10 and the p.Gly1131Valfs*19 and p.Pro929Leu variants were transfected into HEK293 cells. The cells were either untreated (-) or treated (+) with 50 μm MG132 for 24 hours on the third day post-transfection. Lysates were then prepared, fractionated by SDS-PAGE and probed with anti-mCherry and tubulin as a loading control. (**D**) TRAPPC10^-/-^ cells were either untransfected or transfected with FLAG-tagged wild type TRAPPC10, the p.Gly1131Valfs*19 variant or the p.Pro929Leu variant. After 24 hours, lysates were prepared and probed for the FLAG constructs to verify expression, TRAPPC9 and tubulin as a loading control. Parental HEK293 cells were also probed to assess the level of TRAPPC9 in the presence of TRAPPC10. (**E**) Lysates were prepared from lymphoblastoid cells from either control, an individual homozygous for the TRAPPC10 p.Gly1131Valfs*19 variant or three individuals homozygous for the TRAPPC9 p.(Arg475*) variant and subjected to western analysis to reveal the proteins indicated.

We then examined the consequences of TRAPPC10 loss on membrane trafficking. Since we were unable to perform trafficking assays in smaller LCLs, we generated HEK293 TRAPPC10 knockout cell lines using CRISPR/Cas9. Three different TRAPPC10 knockout lines were generated (S3 Fig), two were examined and behaved identically (TRAPPC10^-/-^ KO1/KO7); as such we present data from one line (KO7, hereafter referred to as TRAPPC10^-/-^). We first examined TRAPP protein levels in TRAPPC10^-/-^ cells. Consistent with observations in LCLs, we noted a slight reduction in core TRAPP proteins, no effect on TRAPP III-specific proteins (TRAPPC8, TRAPPC12), and the absence of TRAPPC9, consistent with size exclusion chromatography results (Fig 2E). We then performed the VSVG-GFP ts045 trafficking assay [28] in TRAPPC10^-/-^ cells. In this assay, the marker protein VSVG-GFP ts045 is retained in the ER at elevated temperature, but synchronously released at 32°C. The fluorescently-tagged marker protein accumulated in the Golgi region in controls, with peak signal at ~25 minutes (Fig 3A, black curve). From that point onward the signal was cleared from the Golgi as the protein travelled to the plasma membrane. In contrast, the arrival into, trafficking through and release from the Golgi of the marker protein in TRAPPC10^-/-^ cells was delayed compared to controls (Fig 3A, green curve). Importantly, the trafficking delay was shown to be TRAPPC10-dependent as cell transfection with wild type (WT) TRAPPC10 resulted in a trafficking defect rescue (Fig 3A, blue curve). Although both p.Gly1131Valfs*19 and p.Pro929Leu variants were able to partially rescue these defects, neither rescued anterograde trafficking to the same extent as WT protein (Fig 3A, red/grey curves). Taken together, these results demonstrate that the *TRAPPC10* variants cause partial functional defects, and that the absence of TRAPPC10 results in a concomitant absence of TRAPPC9.

### Expression of TRAPPC10 is important for the detection of a cellular pool of TRAPPC9

As no antibody was available to determine whether mutant TRAPPC10 p.(Gly1131Valfs*19) polypeptide may be expressed in affected individuals, we generated WT and both (p.Gly1131Valfs*19, p.Pro929Leu) constructs to investigate protein stability. The constructs were transfected into HEK293 cells and lysates prepared daily for four days. As shown in Fig 3B, WT protein levels on day 4 dropped to ~70% of that on day 1. In contrast, levels of both altered TRAPPC10 proteins (day 4) dropped to 25–50% (day 1), with the drop in the p.Gly1131Valfs*19 protein being most dramatic. The decrease in expression of WT and both p.Gly1131Valfs*19 and p.Pro929Leu TRAPPC10 proteins was blocked by inclusion of proteasome inhibitor MG132 (Fig 3C). These results suggest that the two mutant proteins are subject to enhanced degradation compared to WT protein.

The absence of TRAPPC9 in TRAPPC10 p.(Gly1131Valfs*19) LCLs prompted us to closely examine the inter-relationship between TRAPPC9 and TRAPPC10. We first investigated whether TRAPPC10 expression in TRAPPC10^-/-^ cells would restore TRAPPC9 levels. Upon transfection of WT TRAPPC10 into TRAPPC10^-/-^ cells, the appearance of TRAPPC9 was noted, although not to parental HEK293 cell levels (Fig 3D). Overexpression of the p.Pro929Leu variant, and to a much lesser degree p.Gly1131Valfs*19, restored some TRAPPC9 protein although again not to the same extent as WT TRAPPC10.

We next investigated whether a reciprocal absence of TRAPPC10 occurred in cells devoid of TRAPPC9. We probed LCL lysates from three individuals with the p.(Arg475*) TRAPPC9 variant [19]. As expected TRAPPC9 was not detected in these cells (Fig 3E), nor was it detected in TRAPPC10 p.(Gly1131Valfs*19) cells (as shown above). Importantly, while TRAPPC10 levels were significantly reduced in p.(Arg475*) TRAPPC9 lysates, the protein was nevertheless present. Collectively, these results suggest that the detection of a cellular pool of TRAPPC9 is strongly dependent upon the expression of full length TRAPPC10.

### *Trappc10*^-/-^ mice have similar neurodevelopmental deficits to patients with biallelic *TRAPPC10* variants

We then evaluated tissue sections and datasets from a *Trappc10*^*tm1b(EUCOMM)Wtsi*^ mouse model, with a focus on the phenotypical components of the human disorder in particular neuroanatomical findings. At weaning age, mouse survival was evaluated from successfully genotyped mice originating from multiple litters derived from a heterozygous-by-heterozygous breeding scheme. We obtained the expected number of WT, heterozygous and homozygous mice. Male and female mice were weighed the same day each week from 4 until 16 weeks of age (S4 Fig).

**Fig 4:**
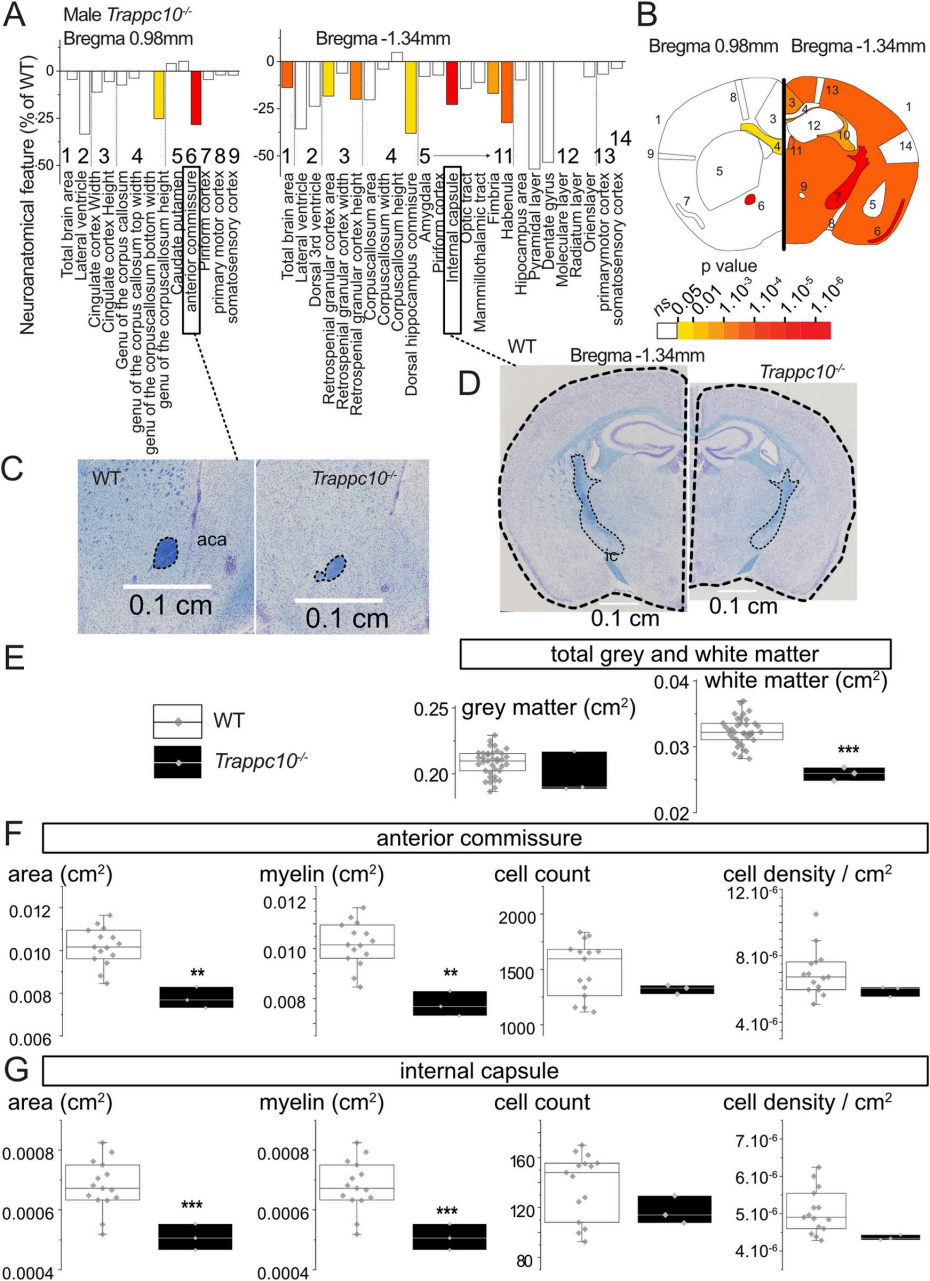
Mouse studies implicate TRAPPC10 in the formation of white matter structures. (**A**) Histograms for three homozygous *Trappc10*^-/-^ mice showing variation (decreased-minus scale or increased-positive scale) in areas and lengths expressed as percentage of 498 WTs together with a color map indicating the significance level (white indicates not significant). The list of measurements and corresponding numbers are shown below the histograms. (**B**) Schematic representation of a section at Bregma +0.98 mm and Bregma -1.34 mm. Colored regions indicate the presence of at least one significant parameter within the brain region at the 0.05 level. (**C-D**) Nissl-stained sagittal brain sections from *Trappc10*^*tm1b/tm1b*^ mice, showing the anterior commissure (**C**) and the total brain area and internal capsule size reduction in mutants (**D**). The corresponding scale is shown in each panel. (**E**) Box plots of combined grey and white matter structures expressed in cm^2^ using a set of 37 local WTs (same genetic background, housing conditions, age, sex, experimental and necropsy dates) compared to three *Trappc10*^*tm1b/tm1b*^ mice. (**F-G**) Box plots with raw data points detailing the cellular and myelination characterisation of the anterior commissure (**F**) and internal capsule (**G**) using a set of 15 local WTs. Statistical analyses were performed with GraphPad Prism 8.0.2, using two-tailed Student’s *t*-tests of equal variances. *p<0.05 **p<0.01 ***p<0.001.

Using a recently developed robust approach to assess 63 brain parameters across 23 brain regions [29], we analyzed neuroanatomical defects in adult *Trappc10*^-/-^ mice blinded for genotype. To minimize environmental and genetic variation, male mice aged 16 weeks were used. In the homozygous mutant mice, many neuroanatomical parameters were reduced in size when compared to WTs (Fig 4A and 4B). The total brain area parameter was significantly reduced (14%, P = 0.0003), concomitantly with smaller white matter structure size including the genu of the corpus callosum (-25%, P = 0.026), hippocampal commissure (-38%, P = 0.013), hippocampal fimbria (-17%, P = 0.0049), anterior commissure (-28%, P = 3.8E10^-8^) and the internal capsule (-23%, P = 1.1E10^-6^) (Fig 4C and 4D). When combining neuroanatomical parameters from either grey or white matter structures, only white matter structures were reduced in size (-20%, P = 8E10^-6^) (Fig 4E). Interestingly, the size reductions of the anterior commissure and internal capsule were correlated with loss of myelination whilst oligodendrocyte cell population count and density were unaffected (Fig 4F and 4G). Together these findings suggest that both mouse and human microcephaly stems from defects in white matter structures and myelin biogenesis.

Given the short stature exhibited by patients with *TRAPPC10*-related disorder we also evaluated *Trappc10*^*-/-*^ mice growth parameters (S1 Text). While long bone length was marginally reduced in both sexes, it only reached significance for female *Humerus* (S4A Fig), although body length overall was unaffected (S4B Fig). While weight was unaffected in *Trappc10*^*-/-*^ males, female *Trappc10*^*-/-*^ mice were overweight, showing a rapid weight increase commencing week 10 (S4C Fig). We investigated the cause of the female obesity phenotype and noted a highly significant fat mass increase (P = 9E-08; S4D Fig), alongside a blood cholesterol increase (P = 4E-05) and high-density lipoprotein levels (P = 0.003) (S4E Fig).

We finally sought to further characterize the phenotypical relationship between *Trappc10*^*-/-*^ mice, and a previously published *Trappc9*^*-/-*^ mouse model [30]. Due to the unavailability of live *Trappc9*^*-/-*^ animals, these evaluations were limited to assessments undertaken on historical brain tissue sections. Overall, the impact of TRAPPC10 loss on brain anatomy was more severe than TRAPPC9 loss (S5 Fig). While there were some overlaps between affected brain regions (e.g. corpus callosum reduced size), some neuroanatomical phenotypes were specific to *Trappc10*^*-/-*^ including reduced size of the anterior commissure.

## Discussion

Here, we define biallelic *TRAPPC10* variants as a cause of a neurodevelopmental TRAPPopathy disorder, with core clinical features including microcephaly, severe global developmental delay and intellectual disability, short stature and pervasive behavioural abnormalities (Table 1). Additional variable features include hypotonia, gait abnormalities and seizures. The overlapping phenotypical nature of the TRAPPopathies suggests a common pathomolecular basis of disease involving disruption of TRAPP II/III complex functions. The clinical features of *TRAPPC10*-related disorder also show extensive overlap with many other Golgipathy disorders [25,26] indicative of a common disease mechanism relating to disrupted Golgi trafficking processes. We observed notable phenotypic overlap with *Trappc10*^*-/-*^ mice which display microcephaly, reduced size of white matter brain structures with hypomyelination and skeletal involvement. Brain abnormalities in mice appear to be more severe than those in humans, in which neuroimaging identified profound thinning of the corpus callosum but no other white matter abnormalities or hypomyelination. However as neuroimaging was only available on a single affected individual who appears to display the least severe phenotype of those assessed, it is possible that more extensive abnormalities may be present in other affected individuals who display a greater degree of microcephaly.

Our genetic studies in combination with our functional investigations on both *TRAPPC10^-/-^* and patient-derived cells indicate that TRAPPC10 loss of function likely underlies the clinical phenotype. Previous subcellular trafficking studies of TRAPPC10 depleted COS-7 [3] and HEK293 [4] cells determined that TRAPPC10 mediates early Golgi (although not endoplasmic reticulum) trafficking, revealing disrupted Golgi architecture and vesicular accummulation indicative of an anterograde trafficking defect. Our cell model membrane trafficking assays are in complete alignment with such a TRAPPC10 molecular role, defining delayed Golgi trafficking associated with gene KO which importantly could be rescued, though not completely, by WT, and to a lesser extent by mutant TRAPPC10. These findings may in part be explained by differing outcomes of the *TRAPPC10* variants, including the possibility that the protein C-terminus plays a specific though as yet unknown post-Golgi secretory pathway role. Interestingly, in previous studies the yeast homologue of TRAPPC10 (Trs130) was rendered conditionally lethal by short truncations in the C-terminal portion of the protein, indicative of a critical functional role of this region [1]. The observed trafficking defects herein may be explained by the loss of GEF activity of TRAPP II towards Rab1 [3], Rab11 [9] and/or Rab18 [4], which alongside other Rab GTPases are key regulators of cellular trafficking by recruiting specific effector proteins to membranes, including lipid droplet homeostasis. Rab18 has specifically been implicated in secretory pathway regulation [31], and *RAB18* variants are associated with a neurodevelopmental disorder phenotypically overlapping *TRAPPC10*-related disorder. Although the specific molecular role of TRAPPC10 in neurological development and function remains unclear, it is tempting to speculate that the predominantly neurological phenoype seen in individuals affected by *TRAPPC10*-related disorder may be due to the relatively high rates of membrane trafficking shown to be required in the brain for processes such as synapse remodeling [32,33].

An important outcome of our findings was that absence of TRAPPC10 is also associated with a concomitant absence of TRAPPC9. Additionally, although other TRAPP III and core specific components were detected, levels of core TRAPPC2L were reduced suggesting an absence of TRAPP II complex, with no effect on TRAPP III complex. These findings indicate that TRAPPC10 is crucial to TRAPP II complex stability and function. Consistent with our findings, careful examination of the data in Li et al (2017) [4], though not commented on, reveals a decrease in the levels of TRAPPC9 protein in TRAPPC10^-/-^ HEK293 cells (see Fig 6D in Ref 4). However, our studies showed TRAPPC10 was still detectable in LCLs lacking TRAPPC9 p.(Arg475*), suggesting that TRAPPC9 may only incorporate into TRAPP II in the presence of TRAPPC10, while the reciprocal is not true. Our overexpression studies of mutant TRAPPC10 provide further insight into this, and in particular identify the expression of full length TRAPPC10 to be of importance for the detection of TRAPPC9 within the TRAPP II complex. Collectively, our results suggest that the absence of the TRAPPC10 C-terminus results in TRAPPC10 protein degradation, and consequent loss of TRAPPC9 (through protein degradation or some other mechanism), leading to the absence of TRAPP II.

The concomitant loss of TRAPPC9 associated with pathogenic *TRAPPC10* variants is reflected in the close phenotypical parity of human *TRAPPC9*- (OMIM 613192) and *TRAPPC10*-related conditions (S1 Table) and with both knockout mouse models. Individuals with *TRAPPC9*-related disorder have postnatal-onset microcephaly [34,35]. While the families reported here did not have any recorded birth OFC measurements, there were no reports of affected children having noticably small head size at birth suggesting microcephaly associated with *TRAPPC10*-related disorder may be postnatal. Head growth trajectory was only available for one child, with two measurements available (2.5 and 4.1 years), that indicate microcephaly is present from early childhood and is not progressive. However it remains unclear whether the brain growth was initially normal and then slowed, or whether microcephaly was present from/before birth. Identification of further affected families will help clarify the nature of the microcephaly in *TRAPPC10*-related disorder, as with *TRAPPC9*-related disorder where this was also unclear initially [19].

Biallelic TRAPPC10 loss of function appears to have a more severe phenotypic impact in mice and humans, compared with biallelic TRAPPC9 loss. In human *TRAPPC10*-related disorder, microcephaly appears to be a relatively consistent feature, (8/10 individuals), as compared to ~60% of TRAPPC9 patients [20,36]. Similarly in mouse models, while the age of onset and progression of microcephaly is unclear, *Trappc10*^*-/-*^ mice display more extensive brain size reduction and loss of white matter structures than *Trappc9*^*-/-*^ mice. These findings may be explained by the concomitant loss of TRAPPC9 observed with TRAPPC10 absence, possibly acting as a double knockout. Obesity is reported in ~50% of individuals with *TRAPPC9*-related disorder and is the only clincial feature not currently observed in individuals with *TRAPPC10*-related disorder. It is important to note that these differences are based on a relatively small sample size, a limitation of this study. Nevertheless, the *Trappc10*^*-/-*^ mice share similar phenotypic outcomes compared to individuals with biallelic *TRAPPC10* variants.

Neuroanatomical studies of *Trappc9*^*-/-*^ mice showed overlapping features with *Trappc10*^*-/-*^ mice including reduced brain size predominantly of white matter structures (S5 Fig) [37], although findings of enlarged striatal size were not identified in *Trappc10*^*-/-*^ mice. TRAPPC9 plays a role in the NF-κB signalling pathway through its interaction with NF-κB inducing kinase (NIK) and the beta subunit of IKK, which both regulate the NF-κB pathway [11,38]. Given the associated loss of TRAPPC9 observed with both the *TRAPPC10* variants and *TRAPPC10^-/-^* cells, it is plausible that TRAPPC10 loss of function may also impact NF-κB signalling pathways through disruption of TRAPPC9, although the involvement of TRAPPC9 in this pathway in brain has been recently questioned [37]. Taken together, we define the genetic, clinical and molecular basis of a novel microcephalic neurodevelopmental disorder associated with biallelic *TRAPPC10* variants in both humans and mice. Our data provides a molecular rationale for the phenotypic overlap between *TRAPPC10*- and *TRAPPC9*-related TRAPPopathy disorders, both involving disruption of TRAPP II-mediated post-Golgi trafficking processes.

## Material and methods

### Ethics statement

This study was carried out in accordance with institutional ethics review board-approved research protocols from the Institutional Review Board, International Islamic University (IIU), Islamabad, Pakistan. All individuals (or their families) whose data is included in this study provided written informed consent and where applicable specific written consent for publication of photographs.

### Genetic studies

Genomic DNA was extracted from blood/buccal samples using the ReliaPrep kit (Blood gDNA Miniprep System, Promega) and RNA was extracted using the PAXgene blood miRNA kit and converted to cDNA via reverse transcriptase PCR. Single nucleotide polymorphism (SNP) genotyping was performed using Illumina HumanCytoSNP-12 v2.1 beadchip array, as previously described [39]. WES analysis was performed (NextSeq500; Illumina, San Diego, CA, USA) and involved Agilent Sureselect Whole Exome v6 (Agilent Technologies, Santa Clara, CA) targeting, read alignment (BWA-MEM v0.7.17), mate-pairs fixed and duplicates removed (Picard v2.15), InDel realignment and base quality recalibration (GATK v3.7.0), single nucleotide variant and InDel detection (GATK HaplotypeCaller), variant annotation (Alamut batch v1.10 and SnpEff (http://snpeff.sourceforge.net/SnpEff_manual.html))) and read depth assessment (GATK DepthOfCoverage). Copy number variants (CNVs) were detected using SavvyCNV [40]. Variants with <5 reads and/or an allele frequency >0.01 in public genome databases including the Genome Aggregation Database (gnomAD v2.1.1 and v3.1) and the 1000 Genomes Project were excluded. Homozygous and compound heterozygous variants that were exonic and non-synonymous, synonymous with predicted splicing impact or intronic at ±6 nucleotides from splice site were evaluated and cross referenced against the SNP data. Dideoxy sequencing validation of *TRAPPC10* variants was undertaken using standard techniques.

### Molecular studies

#### Construction of *TRAPPC10*^*-/-*^ cell lines

Guide RNA (gRNA) design and cloning (CRISPR plasmid pSPCas9(BB)-2A-Puro (PX459) V2.0, Addgene #62988) was performed as described previously [41], with primers: 5’-CACCGCATCTTCGGAGCCCGGCCAT-3’ and 5’-AAACATGGCCGGGCTCCGAAGATGC-3’. HEK293 cells were transfected (70% confluency) using JetPRIME with 500ng DNA expressing the gRNA. Following selection (puromycin) and colony expansion, genomic DNA was extracted. The *TRAPPC10* gene region of interest was amplified using oligonucleotide pair: 5’-AGCGTAGTTATGATTTGGGGT-3 and 5’-GCCAAGGAATGAAGGGACAA-3. The resulting PCR product (~540bp, agarose gel electrophoresis) was sequenced. Knockout verification was performed by western analysis (S3 Fig).

#### Membrane trafficking assay

Cells were infected with virus expressing VSVG-GFP-tsO45 (one hour, 37°C). In rescue experiments, cells were transfected with either FLAG-TRAPPC10, FLAG-TRAPPC10 p.Gly1131Valfs*19 or FLAG-TRAPPC10 p.Pro929Leu constructs, then infected with VSVG–GFP ts045 (one hour, 37°C). Cells were maintained at 40°C overnight. Cycloheximide was added (final concentration, 10μg/ml), prior to shifting cells to 32°C. Time-lapse microscopy started three minutes after temperature shift to allow time to select fluorescent cells (Nikon inverted confocal microscope) as previously described [22]. Movies used for quantitative fluorescence analysis were not subjected to processing. Integrated fluorescence intensity at the Golgi region (defined by the region of perinuclear intensity seen 20–40 minutes after temperature shift) and from whole cell was measured using ImageJ (measurements obtained every 5 minutes). The ratio between fluorescent intensities within the Golgi region and whole cell was generated for each time point. The kinetics of VSVG-GFP-tsO45 trafficking represent change in that ratio over time (0 to 90 min).

#### Cell lysis and size exclusion chromatography

Cells were grown in either DMEM (for HEK293) or RPMI 1640 (for lymphoblastoid cells) medium containing 10% fetal bovine serum (FBS). Cells were lysed in a solution containing 50mM Tris pH 7.2, 150 mM NaCl, 0.5 mM EDTA, 1 mM DTT, 1% Triton X-100 and protease inhibitor cocktail (EDTA-free; Roche). A total of 2–5 mg of protein was fractionated on a Superose 6 Increase 10/300 GL column at a flow rate of 0.4 ml/min. Fractions of 0.5 ml were collected in wash buffer (50 mM Tris pH 7.2, 150 mM NaCl, 0.5 mM EDTA, 1 mM DTT, 0.1% Triton X-100) and probed with the indicated antibodies.

#### Yeast two hybrid assay

Open reading frames (ORFs) encoding TRAPP proteins were cloned (pGADT7 and pGBKT7 vectors; Clontech). Plasmids were transformed into yeast cells (AH109, Y187). Diploids containing the respective TRAPP ORFs were produced by mating and selecting on synthetic complete medium (SC) lacking leucine and tryptophan. Interactions were assessed on SC medium lacking leucine, tryptophan, histidine and adenine. Serial cell dilutions were spotted onto solid medium. Quantification of interaction was assessed using a β-galactosidase assay employing ONPG and normalized to total protein.

#### Molecular biology techniques and reagents

Standard molecular biological techniques were used to generate FLAG-tagged constructs and the stated *TRAPPC10* variants. Commercially available antibodies used in this study were: anti-TRAPPC2 (rabbit polyclonal, homemade), anti-TRAPPC2L (mouse monoclonal, Santa Cruz sc-377322), anti-TRAPPC3 (rabbit polyclonal, homemade), anti-TRAPPC8 (rabbit polyclonal, Abcam ab122692), anti-TRAPPC9 (rabbit polyclonal, LS Bio LS-C750497), anti-TRAPPC10 (mouse monoclonal, Santa Cruz sc-101259), anti-TRAPPC12 (rabbit polyclonal, homemade), anti-FLAG (mouse monoclonal, Sigma F1804), anti-α-Tubulin (mouse monoclonal, Sigma T6199).

### *Trappc10* knockout mouse model

The mouse model was generated by homologous recombination in embryonic stem cells using the Knockout-first allele method [42], adopting a strategy identifying an exon common to all transcripts (exon 14), upstream of which a LacZ cassette was inserted (S6 Fig). Exon 14 of the *Trappc10* allele, flanked by *loxP* sequences bilaterally, was deleted using a Cre recombinase that recognizes *loxP* sites, producing the *Trappc10*^*tmb(EUCOMM)Wtsi*^ knockout allele. Mice were phenotyped by the Mouse Genetics Project (MGP) pipeline at the Wellcome Sanger Institute, UK (S1 Text).

### Mouse neuroanatomical studies

The use of mice in the Wellcome Sanger Institute study was carried out in accordance with UK Home Office regulations (license number 80/2076), UK Animals (Scientific Procedures) Act of 1986. Neuroanatomical studies were carried out using three homozygous *Trappc10*^-/-^ and 498 baseline WT mice on a C57BL/6N pure genetic background at 16-weeks of age as previously described [29,43]. We used a recognized statistical model (Gpower) validated for comparison of modest numbers (n = 3) of animals to evaluate neuroanatomical defects with an effect size of 10% or more with 80% detection power [43]. Paraffin embedded brain samples were cut at 5μm thickness (sliding microtome, Leica RM 2145) to obtain coronal brain region at Bregma +0.98 mm and Bregma -1.34 mm according to the Allen Mouse Brain Atlas [44]. Sections were stained with 0.1% Luxol Fast Blue (Solvent Blue 38; Sigma-Aldrich) and 0.1% Cresyl violet acetate (Sigma-Aldrich) and scanned (Nanozoomer 2.0HT, C9600 series) at 20× resolution. Sixty-three brain parameters made up of area and length measurements as well as cell level features, were taken across the two coronal sections (S1 Text). Co-variates, for example sample processing dates and usernames, were collected at every step of the procedure and used to identify data drifts. Using in-house ImageJ plugins, an image analysis pipeline was used to standardize measurements of areas and lengths. Images were quality controlled for the accuracy of sectioning relative to the reference atlas and controlled for asymmetries and histological artefacts. All samples were also systematically assessed for cellular ectopia (misplaced neurons). Data were analyzed using a linear mixed model framework to determine whether a brain region is associated with neuroanatomical defect or not.

## Supporting information

S1 Text Supplementary Material and Methods. (DOCX)

S1 Table Clinical features associated with TRAPPopathy disorders. Abbreviations: TRAPP; transport protein particles. XLR; X-linked recessive, AR; autosomal recessive, BMI; body mass index, CK; creatine kinase.
(DOCX)

S1 Fig Clinical features of individuals with biallelic *TRAPPC10* variants. **(A)** Individual 1-IV:2 (Family 1) with no evidence of skeletal disproportion. **(A-C)** Show the facial features of two affected individuals from Family 1 displaying mild craniofacial dysmorphism comprising microcephaly, synophrys and upslanting palpebral fissures.
(TIF)

S2 Fig Chromatogram of *TRAPPC10* c.3392del rtPCR product. Confirmation of the outcome of the c.3392del; p.(Gly1131Valfs*19) *TRAPPC10* gene variant on the RNA transcript in blood of an affected individual (individual IV:2, Family 1), demonstrating that this frameshift variant results in a premature stop codon 19 codons downstream.
(TIF)

S3 Fig Characterization of the *TRAPPC10^-/-^* cell lines in HEK293 cells. Lysate was prepared from parental HEK293 cells and from 3 clones (KO1, KO3 and KO7) that were treated with sgRNA targeting *TRAPPC10*. The lysates were fractionated by SDS-PAGE and probed for TRAPPC10. Tubulin was included as a loading control.
(TIF)

S4 Fig Whole-body phenotype analysis of *Trappc10^-/-^* knockout mouse. **(A)** Bone length for long bones (Femur and Humerus) in both sexes (n = 7 for all groups) **(B)** Body length for male *Trappc10^-/-^* (n = 7) and WT (n = 41) and females *Trappc10^-/-^* (n = 7) and WT (n = 27). **(C)** Body weight curves in grams of male and female *Trappc10*^*-/-*^ mice, between 4 and 16 weeks of age. **(D)** Fat body composition in grams of male and female *Trappc10*^*-/-*^ mice at 16 weeks of age. **(E)** *Left*: List of 27 assessed clinical blood chemistry parameters and association in female *Trappc10*^*-/-*^ mice at 16 weeks of age. *Right*: Box plots with raw data points showing results for levels of total cholesterol and high-density lipoprotein (HDL) in millimoles per liter in female *Trappc10*^*-/-*^ mice at 16 weeks of age. Statistical analyses were performed with GraphPad Prism 8.0.2, using two-tailed Student’s *t*-tests of equal variances. *p<0.05 **p<0.01 ***p<0.001. Arrows indicate directionality of effect and “ns” indicates not significant (p-value>0.05).
(TIFF)

S5 Fig Neuroanatomical assessment of *Trappc9* knockout mouse. **(A)** Histograms of percentage change relative to *Trappc9*^*+/+*^ (set as 0) for each of the measured parameters. **(B)** Schematic representation of the 22 brain regions quantified at lateral +0.60 mm on sagittal section from *Trappc9*^*+/+*^ (n = 4) and *Trappc9*^*-/-*^ (n = 4) mice. Colored regions indicate the presence of at least one significant parameter within the brain region at the 0.05 level. White indicates a p-value > 0.05, grey shows not enough data to calculate a p-value. **(C)** List of assessed brain parameters.
(TIFF)

S6 Fig Allelic construction showing targeting of critical exon 14 in the *Trappc10*^*tm1b(EUCOMM)Wtsi*^ mouse. (TIFF)

S7 Fig Representative images for VSV-G trafficking at 0, 20, 40 and 90 minutes. Wild type HEK293 cells or *TRAPPC10^-/-^* cells either not transfected or transfected with FLAG-tagged wild type TRAPPC10 or one of the *TRAPPC10* variants indicated were infected with VSVG-GFP ts045 4 hours after transfection. After an overnight incubation at 40°C, the cells were shifted to 32°C and imaged every minute. Representative images at 0, 20, 40 and 90 minutes are shown.
(TIF)
