# Supplementary material for: Biallelic variants in TRAPPC10 cause a microcephalic TRAPPopathy disorder in humans and mice
Source: PLoS Genet. 2022 Mar 17;18(3):e1010114. doi: 10.1371/journal.pgen.1010114 (PMC8963566; doi:10.1371/journal.pgen.1010114)
Supplement: S1 Text — (DOCX) [file pgen.1010114.s001.docx]

**Supplementary Material and Methods**

**Mouse Studies**

Animal care was as previously described by White et al. (2013) [1]. Mice were fed a standard chow diet using the autoclavable mouse breeder diet 5021 ([www.labdiet.com](http://www.labdiet.com)). After weaning, animals were housed three to four mice per cage with WT controls housed separately, in a specific pathogen-free environment in individually ventilated cages under 12/12 light/dark cycle with temperature-controlled conditions and free access to food and water with hardwood bedding. All animals were regularly monitored for health and welfare concerns and were additionally checked prior to and after procedures. Mice of both genders were weighed between 4 and 16 weeks of age.

**Mouse neuroanatomical studies**

Mice were anaesthetized with Ketamine (100 mg/kg, intraperitoneally) and Xylazine (10 mg/kg, intraperitoneal), blood collected via the retro-orbital route and death confirmed before the brains were dissected and fixed in 4% buffered formalin for 48 hours, then transferred to 70% ethanol. Samples were embedded in paraffin using an automated embedding machine (Sakura Tissue-Tek VIP).

Sixty-three brain parameters made up of area and length measurements as well as cell level features, were taken across the two coronal section regions at Bregma +0.98 mm and Bregma -1.34 mm. Co-variates, for example sample processing dates and usernames, were collected at every step of the procedure and used to identify data drifts. Using in-house ImageJ plugins, an image analysis pipeline was used to standardize measurements of areas and lengths. Each image was quality controlled for the accuracy of sectioning relative to the reference atlas and controlled for asymmetries and histological artefacts. At Bregma +0.98mm brain structures assessed were: 1) the total brain area, 2) the lateral ventricles, 3) the cingulate cortex, 4) the genu of the corpus callosum, 5) the caudate putamen, 6) the anterior commissure, 7) the piriform cortex, 8) the primary motor cortex and 9) the secondary somatosensory cortex. At Bregma -1.34mm, a maximum of 14 brain structures were assessed: 1) the total brain area, 2) the lateral and third ventricles, 3) the retrosplenial granular cortex, 4) the corpus callosum, 5) the amygdala, 6) the piriform cortex, 7) the internal capsule, 8) the optic tract, 9) the mammillothalamic tract, 10) the fimbria of the hippocampus, 11) the habenular nucleus, 12) the hippocampus, 13) the primary motor cortex and 14) the secondary somatosensory cortex. All samples were also systematically assessed for cellular ectopia (misplaced neurons).

For the neuroanatomical assessment of *Trappc9^-/-^* mice, the brains were cut into halves along the sagittal midline, and embedded separately in paraffin. The section of interest was determined as corresponding to Figure 106 of the Mouse Brain Atlas (Lateral +0.60 mm) [2]. Brains were sectioned to match the defined section at a thickness of 5μm. The staining, scanning, and quality control steps were identical to the coronal procedure used for the neuroanatomical assessment of *Trappc10^-/-^* mice. A total of 40 brain morphological parameters including 25 areas, 14 lengths and one number, were measured for each brain. These parameters encompassed 22 distinct brain regions.

**Mouse body composition and clinical blood chemistry tests**

Non-fasted mice at 16 weeks of age were terminally anaesthetised with ketamine/xylazine and blood was collected into lithium/heparin coated tubes via the retro-orbital sinus. Plasma was analysed for the following 27 parameters on an Olympus AU400: 1) for electrolytes: sodium, potassium and chloride; 2) for non-fasted metabolic panel: glucose, fructosamine, triglycerides, cholesterol, high-density lipoprotein (HDL), low-density lipoprotein (LDL), non-esterified free fatty acids (NEFAC) and glycerol; 3) for thyroid: total thyroxine and adiponectin; 4) pancreatic enzyme: amylase; 5) liver/muscle: alanine aminotransferase, alkaline phosphatase, creatine kinase, aspartate aminotransferase and total bilirubin; 6) protein parameters: total protein and albumin; 7) kidney: creatinine and urea; and 8) minerals and iron: calcium, magnesium, iron and phosphate. For body composition, 14 week mice were imaged on a dual energy X-ray absorptiometry machine (Lunar PIXImus II). This generated an image of the entire mouse and provided bone mineral, body composition and morphometric data.

1. White JK, Gerdin AK, Karp NA, Ryder E, Buljan M, Bussell JN, et al. Genome-wide generation and systematic phenotyping of knockout mice reveals new roles for many genes. Cell. 2013;154(2):452-64. doi: 10.1016/j.cell.2013.06.022. PubMed PMID: 23870131; PubMed Central PMCID: PMCPMC3717207.

2. Collins SC, Wagner C, Gagliardi L, Kretz PF, Fischer MC, Kessler P, et al. A Method for Parasagittal Sectioning for Neuroanatomical Quantification of Brain Structures in the Adult Mouse. Curr Protoc Mouse Biol. 2018;8(3):e48. Epub 2018/06/26. doi: 10.1002/cpmo.48. PubMed PMID: 29944194.
