# Supplementary material for: Biallelic variants in TRAPPC10 cause a microcephalic TRAPPopathy disorder in humans and mice
Source: PLoS Genet. 2022 Mar 17;18(3):e1010114. doi: 10.1371/journal.pgen.1010114 (PMC8963566; doi:10.1371/journal.pgen.1010114)
Supplement: S1 Table — Abbreviations: TRAPP; transport protein particles. XLR; X-linked recessive, AR; autosomal recessive, BMI; body mass index, CK; creatine kinase. (DOCX) [file pgen.1010114.s002.docx]

| TRAPP subunit | Inheritance | OMIM number and clinical features | References |
| --- | --- | --- | --- |
| TRAPPC2 | XLR | **Spondyloepiphyseal dysplasia tarda (OMIM #313400)**  Short stature, vertebral flattening, kyphosis/scoliosis, small capital femoral epiphyses, mild epiphyseal irregularities | Gedeon et al [1], Fiedler et al [2] |
| TRAPPC2L | AR | **Encephalopathy, progressive, early-onset, with episodic rhabdomyolysis (OMIM #618331)**  Developmental delay, absent speech, microcephaly, epilepsy, regression, rhabdomyolysis, raised CK, tetraplegia, dystonia, cerebral visual impairment | Milev et al [3], Al-Deri et al [4] |
| TRAPPC4 | AR | **Neurodevelopmental disorder with epilepsy, spasticity, and brain atrophy (OMIM #618741)**  Early-onset seizures, developmental delay, microcephaly, sensorineural deafness, spastic quadriparesis, progressive cortical and cerebellar atrophy, scoliosis, facial dysmorphism | Van Bergen et al [5] |
| TRAPPC6A | AR | Intellectual disability, speech delay, facial dysmorphism, polydactyly | Mohamoud et al [6]  Single family described |
| TRAPPC6B | AR | **Neurodevelopmental disorder with microcephaly, epilepsy, and brain atrophy (OMIM #617862)**  Microcephaly, intellectual disability, epilepsy, ataxic gait, cortical atrophy, autistic features, generalised weakness, thin corpus callosum | Marin-Valencia et al [7] |
| TRAPPC9 | AR | **Mental retardation, autosomal recessive 13 (OMIM #613192)**  Microcephaly, intellectual disability, reduced white matter volume, thin corpus callosum, absent speech, hypotonia, dysmorphic features, hyperkinesia, epilepsy, raised BMI | Mortreux et al [8], Mir et al [9], Abbasi et al [10], Mbimba et al [11] |
| TRAPPC10 | AR | Microcephaly, intellectual disability, developmental delay, speech delay, hypotonia, gait abnormalities, epilepsy, autistic features, facial dysmorphism, short stature, strabismus, thin corpus callosum, reduced white matter structures | *This study* |
| TRAPPC11 | AR | **Muscular dystrophy, limb-girdle, autosomal recessive 18 (OMIM #615356)**  Epilepsy, myopathy, microcephaly, developmental delay, ataxia, cerebral atrophy, raised CK, scoliosis, cataracts, hepatomegaly, steatohepatitis, hypotonia, gait abnormalities, short stature, dystonia, spasticity, generalised weakness, achalasia, alacrima | Bogershausen et al [12], Liang et al [13], Koehler et al [14], Milev et al [15], Larson et al [16] |
| TRAPPC12 | AR | **Encephalopathy, progressive, early-onset, with brain atrophy and spasticity (OMIM #617669)**  Hypotonia, microcephaly, epilepsy, global developmental delay, brain atrophy, agenesis of the corpus callosum, pons hypoplasia, spasticity, myoclonic jerks, neurogenic bladder | Milev et al [17] |
| TRAPPC14 | AR | **?Microcephaly 25, primary, autosomal recessive (OMIM #618351)**  Short stature, microcephaly, global developmental delay, impaired intellectual development, speech delay, ADHD, decreased white matter and thin corpus callosum | Perez et al [18]  Single family described |

**S1 Table: Clinical features associated with TRAPPopathy disorders.**

Abbreviations: TRAPP; transport protein particles. XLR; X-linked recessive, AR; autosomal recessive, BMI; body mass index, CK; creatine kinase.

1. Gedeon AK, Tiller GE, Le Merrer M, Heuertz S, Tranebjaerg L, Chitayat D, et al. The molecular basis of X-linked spondyloepiphyseal dysplasia tarda. Am J Hum Genet. 2001;68(6):1386-97. Epub 2001/05/08. doi: 10.1086/320592. PubMed PMID: 11349230; PubMed Central PMCID: PMCPMC1226125.

2. Fiedler J, Le Merrer M, Mortier G, Heuertz S, Faivre L, Brenner RE. X-linked spondyloepiphyseal dysplasia tarda: Novel and recurrent mutations in 13 European families. Hum Mutat. 2004;24(1):103. doi: 10.1002/humu.9254. PubMed PMID: 15221797.

3. Milev MP, Graziano C, Karall D, Kuper WFE, Al-Deri N, Cordelli DM, et al. Bi-allelic mutations in TRAPPC2L result in a neurodevelopmental disorder and have an impact on Rab11 in fibroblasts. J Med Genet. 2018;55(11):753-64. Epub 2018/08/17. doi: 10.1136/jmedgenet-2018-105441. PubMed PMID: 30120216.

4. Al-Deri N, Okur V, Ahimaz P, Milev M, Valivullah Z, Hagen J, et al. A novel homozygous variant in *TRAPPC2L* results in a neurodevelopmental disorder and disrupts TRAPP complex function. J Med Genet. 2020. Epub 2020/08/25. doi: 10.1136/jmedgenet-2020-107016. PubMed PMID: 32843486.

5. Van Bergen NJ, Guo Y, Al-Deri N, Lipatova Z, Stanga D, Zhao S, et al. Deficiencies in vesicular transport mediated by TRAPPC4 are associated with severe syndromic intellectual disability. Brain. 2020;143(1):112-30. doi: 10.1093/brain/awz374. PubMed PMID: 31794024; PubMed Central PMCID: PMCPMC6935753.

6. Mohamoud HS, Ahmed S, Jelani M, Alrayes N, Childs K, Vadgama N, et al. A missense mutation in TRAPPC6A leads to build-up of the protein, in patients with a neurodevelopmental syndrome and dysmorphic features. Sci Rep. 2018;8(1):2053. Epub 2018/02/01. doi: 10.1038/s41598-018-20658-w. PubMed PMID: 29391579; PubMed Central PMCID: PMCPMC5794855.

7. Marin-Valencia I, Novarino G, Johansen A, Rosti B, Issa MY, Musaev D, et al. A homozygous founder mutation in TRAPPC6B associates with a neurodevelopmental disorder characterised by microcephaly, epilepsy and autistic features. J Med Genet. 2018;55(1):48-54. Epub 2017/06/16. doi: 10.1136/jmedgenet-2017-104627. PubMed PMID: 28626029; PubMed Central PMCID: PMCPMC6056005.

8. Mortreux J, Busa T, Germain DP, Nadeau G, Puechberty J, Coubes C, et al. The role of CNVs in the etiology of rare autosomal recessive disorders: the example of TRAPPC9-associated intellectual disability. Eur J Hum Genet. 2018;26(1):143-8. Epub 2017/11/29. doi: 10.1038/s41431-017-0018-x. PubMed PMID: 29187737; PubMed Central PMCID: PMCPMC5838970.

9. Mir A, Kaufman L, Noor A, Motazacker MM, Jamil T, Azam M, et al. Identification of mutations in TRAPPC9, which encodes the NIK- and IKK-beta-binding protein, in nonsyndromic autosomal-recessive mental retardation. Am J Hum Genet. 2009;85(6):909-15. doi: 10.1016/j.ajhg.2009.11.009. PubMed PMID: 20004765; PubMed Central PMCID: PMCPMC2790571.

10. Abbasi AA, Blaesius K, Hu H, Latif Z, Picker-Minh S, Khan MN, et al. Identification of a novel homozygous TRAPPC9 gene mutation causing non-syndromic intellectual disability, speech disorder, and secondary microcephaly. Am J Med Genet B Neuropsychiatr Genet. 2017;174(8):839-45. Epub 2017/10/14. doi: 10.1002/ajmg.b.32602. PubMed PMID: 29031008.

11. Mbimba T, Hussein NJ, Najeed A, Safadi FF. TRAPPC9: Novel insights into its trafficking and signaling pathways in health and disease (Review). Int J Mol Med. 2018;42(6):2991-7. Epub 2018/09/21. doi: 10.3892/ijmm.2018.3889. PubMed PMID: 30272317.

12. Bögershausen N, Shahrzad N, Chong JX, von Kleist-Retzow JC, Stanga D, Li Y, et al. Recessive TRAPPC11 mutations cause a disease spectrum of limb girdle muscular dystrophy and myopathy with movement disorder and intellectual disability. Am J Hum Genet. 2013;93(1):181-90. Epub 2013/07/03. doi: 10.1016/j.ajhg.2013.05.028. PubMed PMID: 23830518; PubMed Central PMCID: PMCPMC3710757.

13. Liang WC, Zhu W, Mitsuhashi S, Noguchi S, Sacher M, Ogawa M, et al. Congenital muscular dystrophy with fatty liver and infantile-onset cataract caused by TRAPPC11 mutations: broadening of the phenotype. Skelet Muscle. 2015;5:29. Epub 2015/08/28. doi: 10.1186/s13395-015-0056-4. PubMed PMID: 26322222; PubMed Central PMCID: PMCPMC4551700.

14. Koehler K, Milev MP, Prematilake K, Reschke F, Kutzner S, Jühlen R, et al. A novel *TRAPPC11* mutation in two Turkish families associated with cerebral atrophy, global retardation, scoliosis, achalasia and alacrima. J Med Genet. 2017;54(3):176-85. Epub 2016/10/05. doi: 10.1136/jmedgenet-2016-104108. PubMed PMID: 27707803.

15. Milev MP, Stanga D, Schänzer A, Nascimento A, Saint-Dic D, Ortez C, et al. Characterization of three TRAPPC11 variants suggests a critical role for the extreme carboxy terminus of the protein. Sci Rep. 2019;9(1):14036. Epub 2019/10/01. doi: 10.1038/s41598-019-50415-6. PubMed PMID: 31575891; PubMed Central PMCID: PMCPMC6773699.

16. Larson AA, Baker PR, Milev MP, Press CA, Sokol RJ, Cox MO, et al. TRAPPC11 and GOSR2 mutations associate with hypoglycosylation of α-dystroglycan and muscular dystrophy. Skelet Muscle. 2018;8(1):17. Epub 2018/05/31. doi: 10.1186/s13395-018-0163-0. PubMed PMID: 29855340; PubMed Central PMCID: PMCPMC5984345.

17. Milev MP, Grout ME, Saint-Dic D, Cheng YH, Glass IA, Hale CJ, et al. Mutations in TRAPPC12 Manifest in Progressive Childhood Encephalopathy and Golgi Dysfunction. Am J Hum Genet. 2017;101(2):291-9. doi: 10.1016/j.ajhg.2017.07.006. PubMed PMID: 28777934; PubMed Central PMCID: PMCPMC5544387.

18. Perez Y, Bar-Yaacov R, Kadir R, Wormser O, Shelef I, Birk OS, et al. Mutations in the microtubule-associated protein MAP11 (C7orf43) cause microcephaly in humans and zebrafish. Brain. 2019;142(3):574-85. doi: 10.1093/brain/awz004. PubMed PMID: 30715179; PubMed Central PMCID: PMCPMC6391606.
