# Supplementary figures and images for: Biallelic variants in TRAPPC10 cause a microcephalic TRAPPopathy disorder in humans and mice
Source: PLoS Genet. 2022 Mar 17;18(3):e1010114. doi: 10.1371/journal.pgen.1010114 (PMC8963566; doi:10.1371/journal.pgen.1010114)

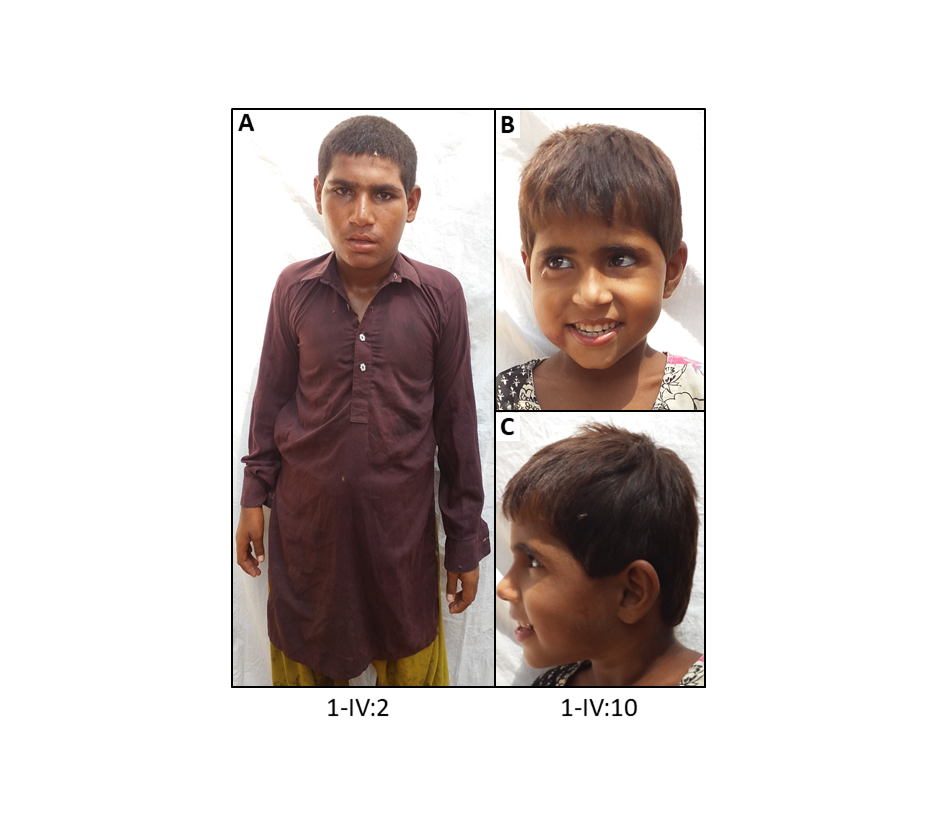

Supplement: S1 Fig — (A) Individual 1-IV:2 (Family 1) with no evidence of skeletal disproportion. (A-C) Show the facial features of two affected individuals from Family 1 displaying mild craniofacial dysmorphism comprising microcephaly, synophrys and upslanting palpebral fissures. (TIF) [file pgen.1010114.s003.tif]

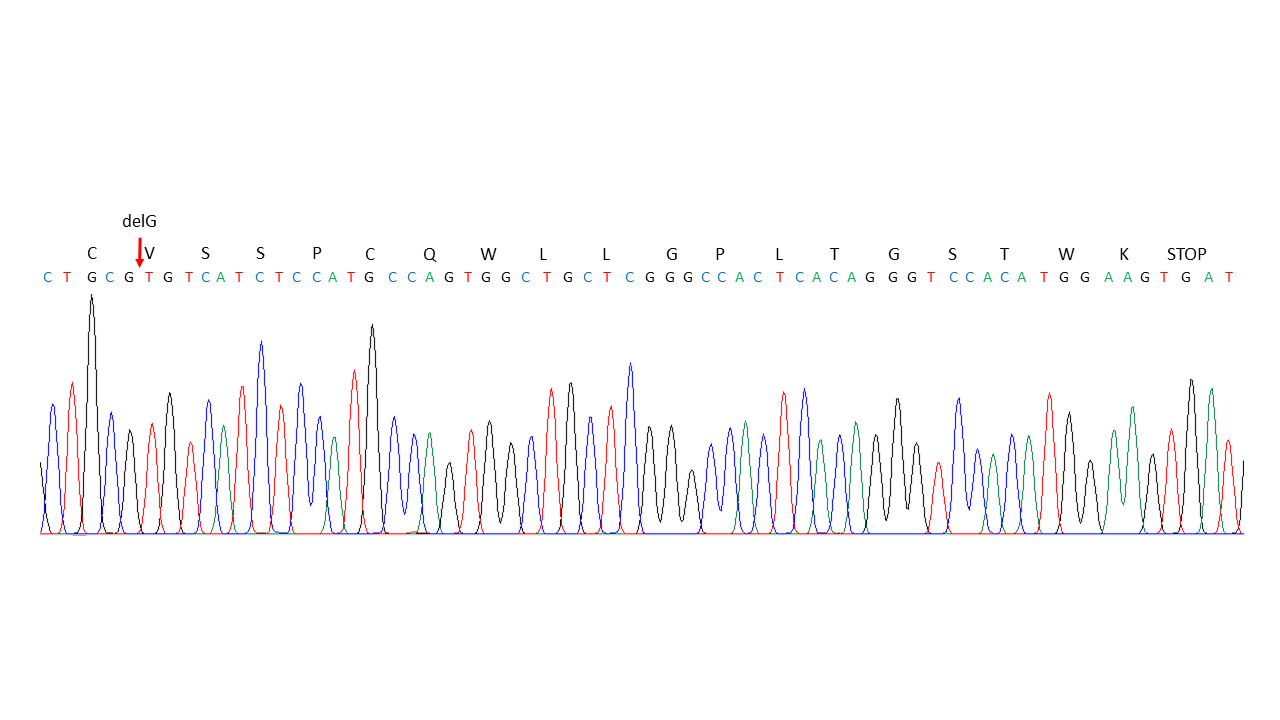

Supplement: S2 Fig — Confirmation of the outcome of the c.3392del; p.(Gly1131Valfs*19) TRAPPC10 gene variant on the RNA transcript in blood of an affected individual (individual IV:2, Family 1), demonstrating that this frameshift variant results in a premature stop codon 19 codons downstream. (TIF) [file pgen.1010114.s004.tif]

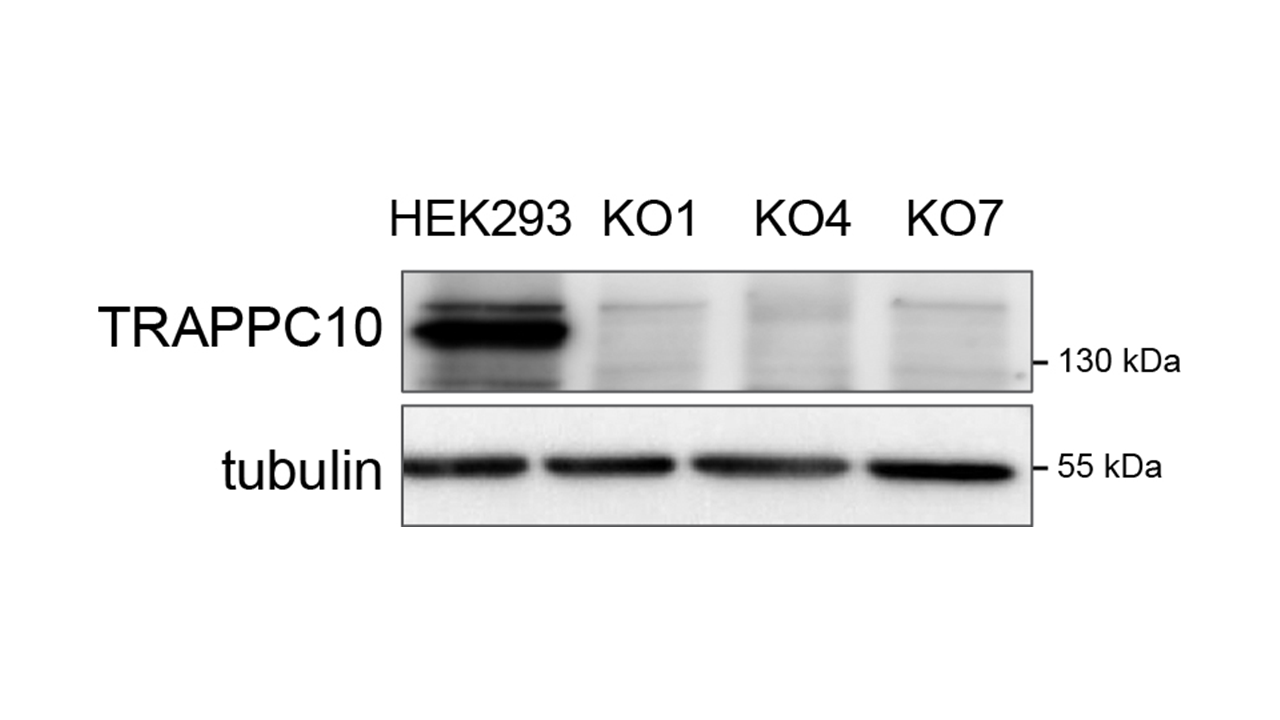

Supplement: S3 Fig — Lysate was prepared from parental HEK293 cells and from 3 clones (KO1, KO3 and KO7) that were treated with sgRNA targeting TRAPPC10. The lysates were fractionated by SDS-PAGE and probed for TRAPPC10. Tubulin was included as a loading control. (TIF) [file pgen.1010114.s005.tif]

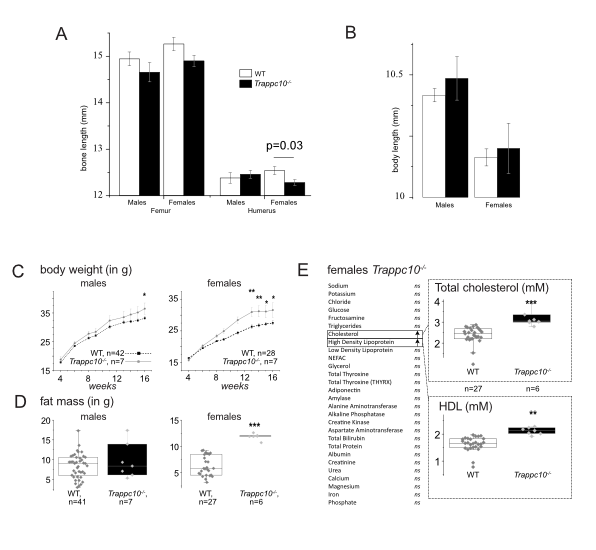

Supplement: S4 Fig — (A) Bone length for long bones (Femur and Humerus) in both sexes (n = 7 for all groups) (B) Body length for male Trappc10-/- (n = 7) and WT (n = 41) and females Trappc10-/- (n = 7) and WT (n = 27). (C) Body weight curves in grams of male and female Trappc10-/- mice, between 4 and 16 weeks of age. (D) Fat body composition in grams of male and female Trappc10-/- mice at 16 weeks of age. (E) Left: List of 27 assessed clinical blood chemistry parameters and association in female Trappc10-/- mice at 16 weeks of age. Right: Box plots with raw data points showing results for levels of total cholesterol and high-density lipoprotein (HDL) in millimoles per liter in female Trappc10-/- mice at 16 weeks of age. Statistical analyses were performed with GraphPad Prism 8.0.2, using two-tailed Student’s t-tests of equal variances. *p<0.05 **p<0.01 ***p<0.001. Arrows indicate directionality of effect and “ns” indicates not significant (p-value>0.05). (TIFF) [file pgen.1010114.s006.tiff]

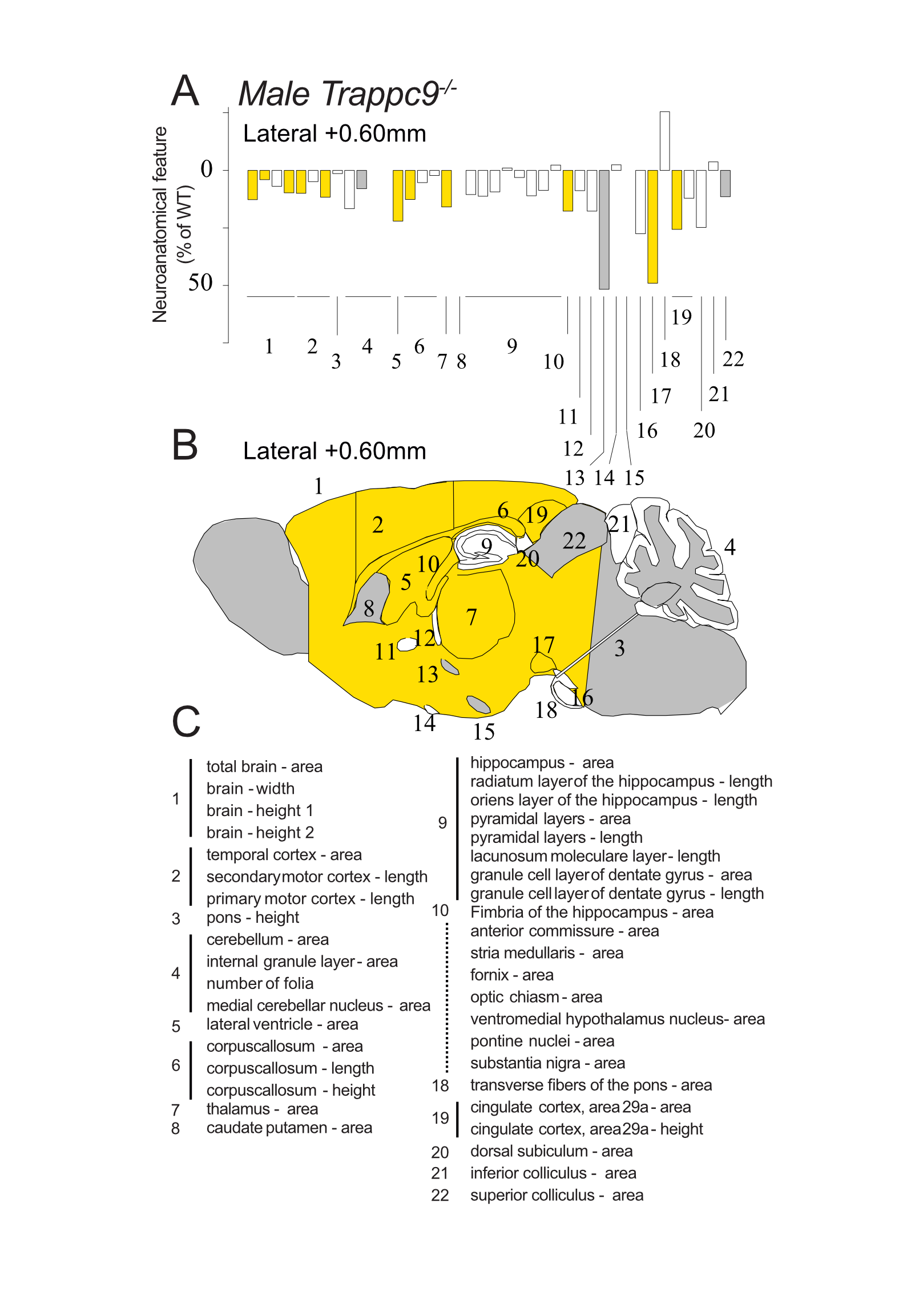

Supplement: S5 Fig — (A) Histograms of percentage change relative to Trappc9+/+ (set as 0) for each of the measured parameters. (B) Schematic representation of the 22 brain regions quantified at lateral +0.60 mm on sagittal section from Trappc9+/+ (n = 4) and Trappc9-/- (n = 4) mice. Colored regions indicate the presence of at least one significant parameter within the brain region at the 0.05 level. White indicates a p-value > 0.05, grey shows not enough data to calculate a p-value. (C) List of assessed brain parameters. (TIFF) [file pgen.1010114.s007.tiff]

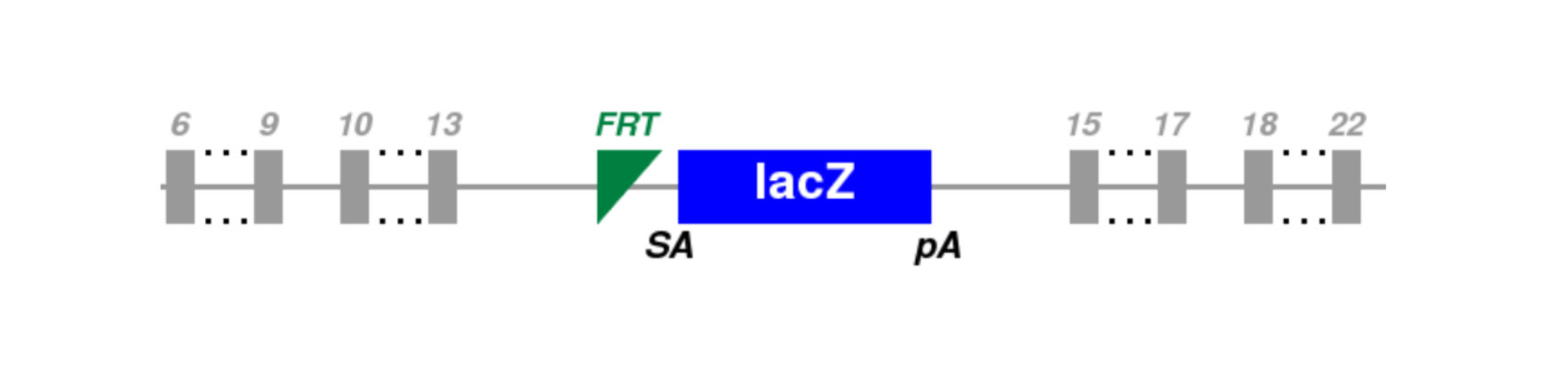

Supplement: S6 Fig — (TIFF) [file pgen.1010114.s008.tiff]

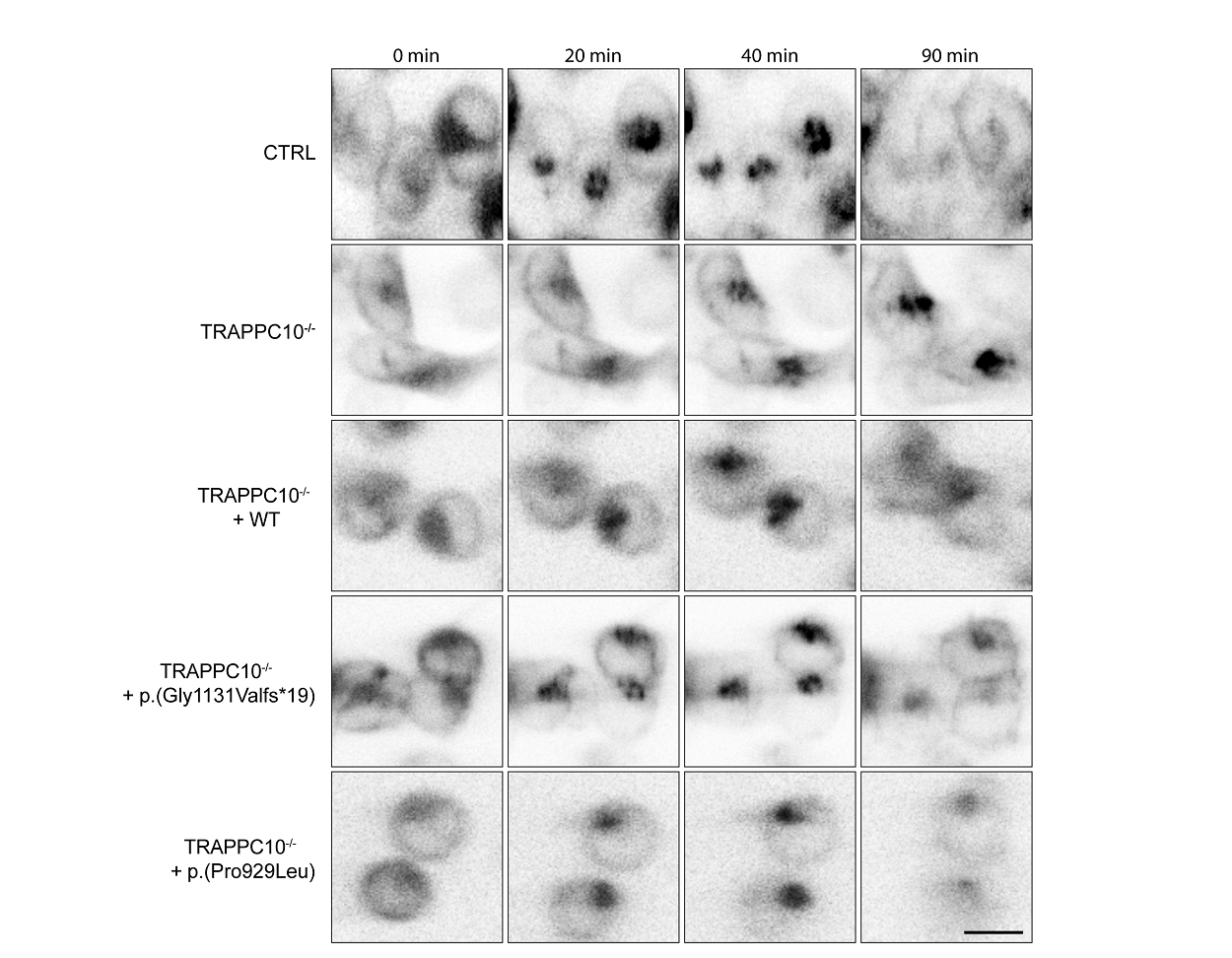

Supplement: S7 Fig — Wild type HEK293 cells or TRAPPC10-/- cells either not transfected or transfected with FLAG-tagged wild type TRAPPC10 or one of the TRAPPC10 variants indicated were infected with VSVG-GFP ts045 4 hours after transfection. After an overnight incubation at 40°C, the cells were shifted to 32°C and imaged every minute. Representative images at 0, 20, 40 and 90 minutes are shown. (TIF) [file pgen.1010114.s009.tif]
